# Supplementary material for: Biomimetic non-classical crystallization drives hierarchical structuring of efficient circularly polarized phosphors
Source: Nat Commun. 2022 Jun 9;13:3339. doi: 10.1038/s41467-022-30989-y (PMC9184729; doi:10.1038/s41467-022-30989-y)
Supplement: Supplementary file 1 — Supplementary information [file 41467_2022_30989_MOESM1_ESM.pdf]

Supplementary Information

Biomimetic non-classical crystallization drives hierarchical structuring of efficient circularly polarized phosphors

Li-Zhe Feng et al.

Supplementary Note 1 Characterization data of organic compounds

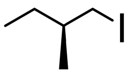

(S)-1-iodo-2-methylbutane

^{1}H NMR (400 MHz, Chloroform-*d*) δ 3.23 (dd, *J* = 9.6, 4.6 Hz, 1H), 3.16 (dd, *J* = 9.6, 5.8 Hz, 1H), 1.45 – 1.35 (m, 2H), 1.29 – 1.23 (m, 1H), 0.97 (d, *J* = 6.5 Hz, 3H), 0.89 (t, *J* = 7.3 Hz, 3H). **^{13}C NMR (101 MHz, Chloroform-*d*)** δ 36.40, 29.20, 20.18, 17.46, 11.31. **HRMS** (ESI) m/z: $(M+H)^+$ calcd. for $C_5H_{12}I^+$, 198.9978; found, 198.9937. **$[\alpha]^{20}_D$** = 0.01 (c = 1, $CHCl_3$).

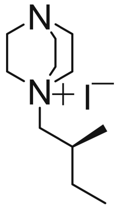

(S)-1-(2-methylbutyl)-1,4-diazabicyclo[2.2.2]octan-1-ium (L_1, (S)-2-Me-bu-ted) iodide

^{1}H NMR (400 MHz, DMSO-d_6) δ 3.36 – 3.29 (m, 6H), 3.19 – 3.12 (m, 2H), 3.02 (t, *J* = 7.5 Hz, 6H), 2.11 (s, 1H), 1.45 – 1.34 (m, 1H), 1.32 – 1.23 (m, 1H), 1.02 (d, *J* = 6.8 Hz, 3H), 0.93 – 0.87 (m, 3H). **^{13}C NMR (101 MHz, DMSO-d_6)** δ 70.13, 52.18, 45.26, 29.34, 27.98, 20.43, 11.31. **HRMS** (ESI) m/z: $(M+H)^+$ calcd. for $C_{11}H_{24}IN_2^+$, 311.0979; found, 311.1014. **$[\alpha]^{20}_D$** = 0.05 (c = 1, CH_3OH).

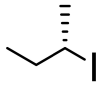

(R)-2-iodobutane

^{1}H NMR (400 MHz, Chloroform-*d*) δ 4.23 – 4.10 (m, 1H), 1.91 (d, *J* = 6.8 Hz, 3H), 1.88 – 1.75 (m, 1H), 1.68 (dqd, *J* = 14.4, 7.2, 5.0 Hz, 1H), 1.00 (t, *J* = 7.2 Hz, 3H). **^{13}C NMR (101 MHz, Chloroform-*d*)** δ 36.00, 32.85, 28.53, 14.24. **HRMS** (ESI) m/z: $(M+H)^+$ calcd. for $C_4H_{10}I^+$, 184.9982; found, 184.9902. **$[\alpha]^{20}_D$** = -0.04 (c = 1, $CHCl_3$).

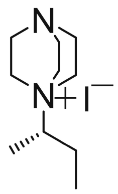

(S)-1-(sec-butyl)-1,4-diazabicyclo[2.2.2]octan-1-ium (L_2, (S)-1-Me-pr-ted) iodide

^{1}H NMR (400 MHz, DMSO-d6) δ 3.35 – 3.18 (m, 7H), 3.01 (t, J = 7.8 Hz, 6H), 2.10 – 1.94 (m, J = 5.6 Hz, 1H), 1.40 – 1.29 (m, 1H), 1.26 (d, J = 6.6 Hz, 3H), 0.93 (t, J = 7.3 Hz, 3H). **^{13}C NMR (101 MHz, DMSO-*d*$_6$)** δ 70.66 (t, *J* = 2.3 Hz), 49.03 – 48.89 (m), 44.83, 21.86, 12.36, 11.14. **HRMS** (ESI) m/z: $(M+H)^+$ calcd. for $C_{10}H_{22}IN_2^+$, 297.0822; found, 297.0865. **$[\alpha]^{20}_D$** = 0.05 (c = 1, CH_3OH).

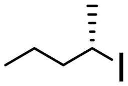

(R)-2-iodoheptane

^{1}H NMR (400 MHz, Chloroform-*d*) δ 4.20 (dqd, *J* = 8.6, 6.8, 4.7 Hz, 1H), 1.92 (d, *J* = 6.8 Hz, 3H), 1.89 – 1.78 (m, 1H), 1.62 – 1.47 (m, 2H), 1.44 – 1.34 (m, 1H), 0.92 (t, *J* = 7.2 Hz, 3H). **^{13}C NMR (101 MHz, Chloroform-*d*)** δ 44.98, 30.46, 28.94, 22.96, 13.16. **HRMS** (ESI) m/z: $(M+Na)^+$ calcd. for $C_5H_{11}INa^+$, 220.9803; found, 220.9813. **$[\alpha]^{20}_D$** = -0.11 (c = 1, $CHCl_3$).

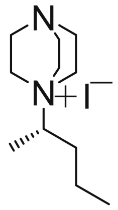

(S)-1-(pentan-2-yl)-1,4-diazabicyclo[2.2.2]octan-1-ium (L_3, (S)-1-Me-bu-ted) iodide

^{1}H NMR (400 MHz, DMSO-*d*$_6$) δ 3.33 – 3.20 (m, 7H), 3.05 – 3.00 (m, 6H), 1.92 (d, *J* = 13.5 Hz, 1H), 1.52 – 1.41 (m, 1H), 1.39 – 1.19 (m, 5H), 0.94 (t, *J* = 7.2 Hz, 3H). **^{13}C NMR (101 MHz, DMSO-*d*$_6$)** δ 69.44, 49.31, 45.24, 31.18, 19.98, 14.18, 13.40. **HRMS** (ESI) m/z: $(M+H)^+$ calcd. for $C_{11}H_{24}IN_2^+$, 311.0979; found, 311.0970. **$[\alpha]^{20}_D$** = 0.02 (c = 1, CH_3OH).

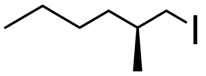

(S)-1-iodo-4-methylhexane

^{1}H NMR (400 MHz, Chloroform-*d*) δ 3.25 – 3.10 (m, 2H), 1.93 – 1.71 (m, 2H), 1.46 – 1.26 (m, 3H), 1.26 – 1.09 (m, 2H), 0.90 – 0.83 (m, 6H). **^{13}C NMR (101 MHz, Chloroform-*d*)** δ 37.42, 33.67, 31.33, 29.33, 19.15, 11.34, 7.59. **HRMS** (ESI) m/z: $(M+H)^+$ calcd. for $C_7H_{16}I^+$, 227.0291; found, 227.0214. **$[\alpha]^{20}_D$** = 0.03 (c = 1, $CHCl_3$).

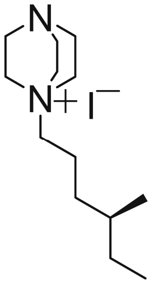

(S)-1-(4-methylhexyl)-1,4-diazabicyclo[2.2.2]octan-1-ium (L_4, (S)-4-Me-hex-ted) iodide

^{1}H NMR (400 MHz, DMSO-*d*$_6$) δ 3.26 (t, *J* = 7.5 Hz, 6H), 3.15 (ddd, *J* = 9.8, 6.2, 2.5 Hz, 2H), 3.02 (dd, *J* = 8.7, 6.1 Hz, 6H), 1.64 (tdd, *J* = 17.0, 10.5, 5.5 Hz, 2H), 1.41 – 1.30 (m, 2H), 1.27 – 1.20 (m, 1H), 1.18 – 1.03 (m, 2H), 0.92 – 0.79 (m, 6H). **^{13}C NMR (101 MHz, DMSO-*d*$_6$)** δ 63.87, 51.95, 45.16, 33.91, 32.93, 29.16, 19.36, 19.27, 11.64. **HRMS** (ESI) m/z: $(M+H)^+$ calcd. for $C_{13}H_{27}IN_2Na^+$, 361.1117; found, 361.1080. **$[\alpha]^{20}_D$** = 0.11 (c = 1, CH_3OH).

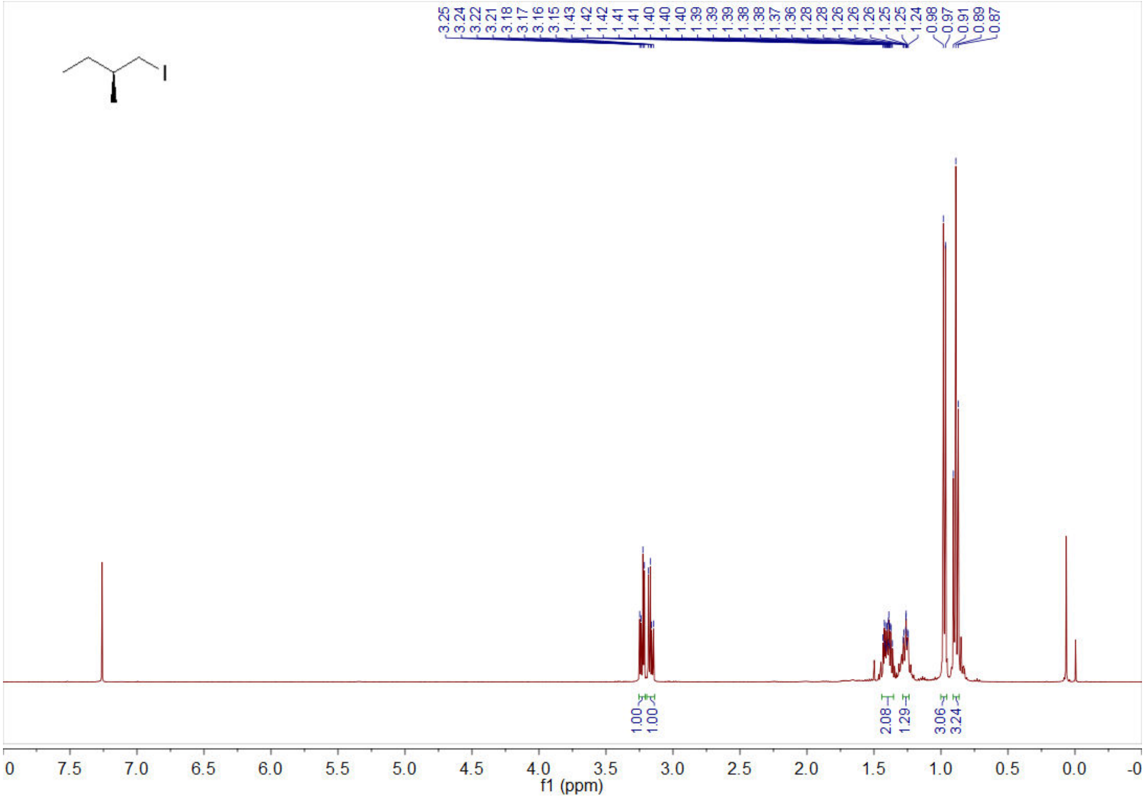

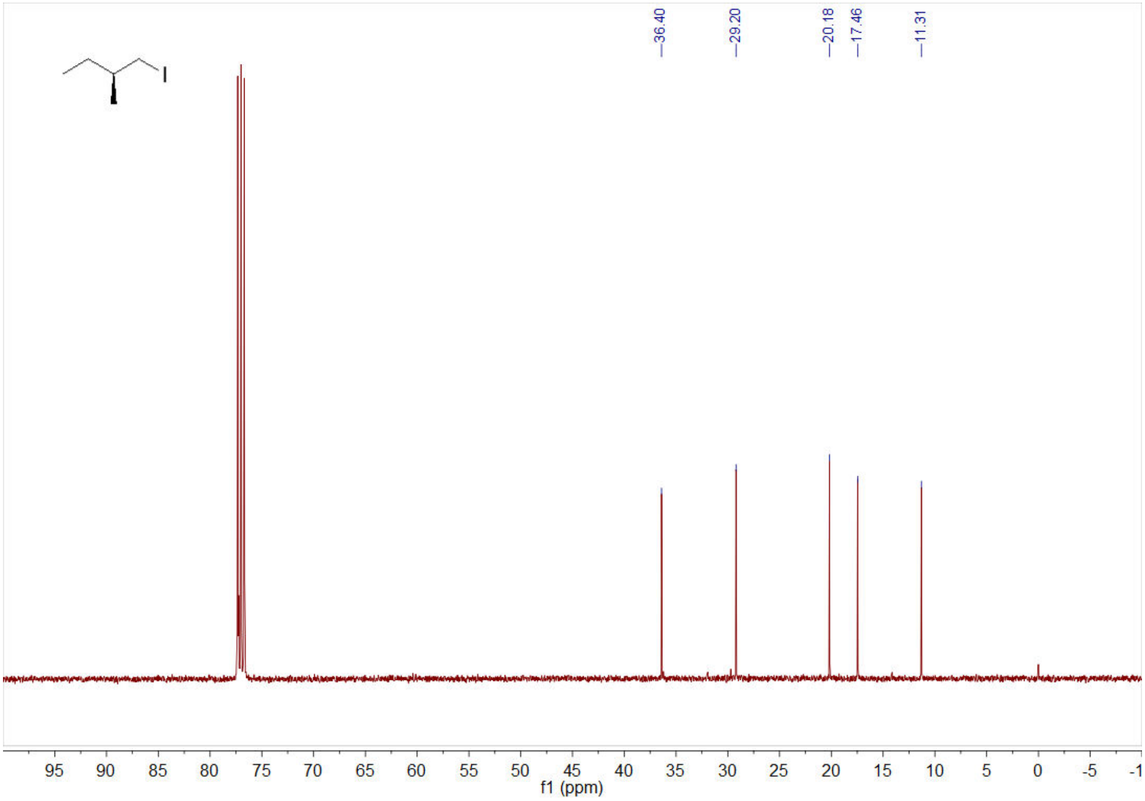

Supplementary Fig. 1. ^{1}H NMR (400 MHz, Chloroform-*d*) and ^{13}C NMR (101MHz, Chloroform-*d*) spectra of (S)-1-iodo-2-methylbutane.

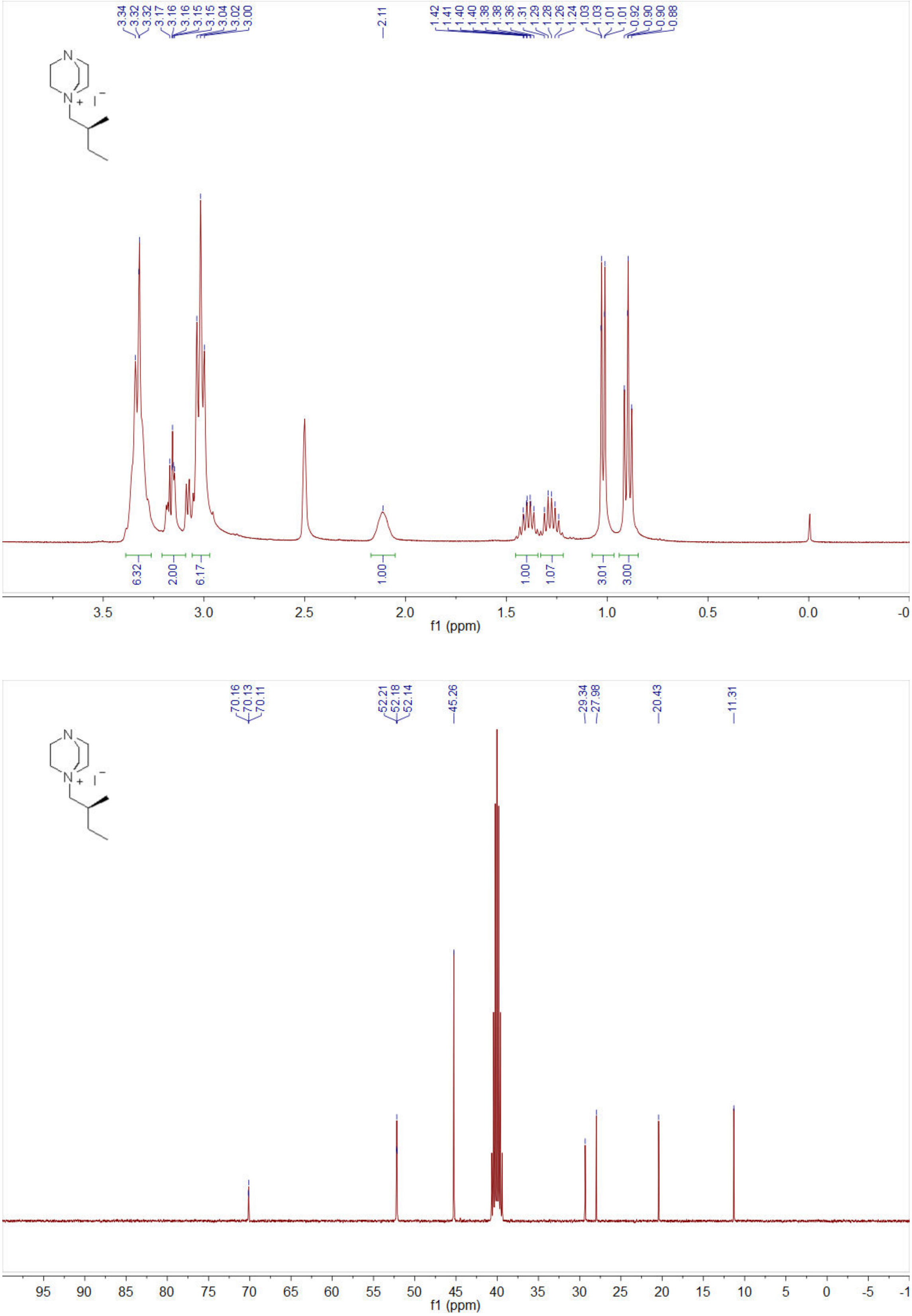

Supplementary Fig. 2. ^{1}H NMR (400 MHz, DMSO-d_6) and ^{13}C NMR (101MHz, DMSO-d_6) spectra of L_1.

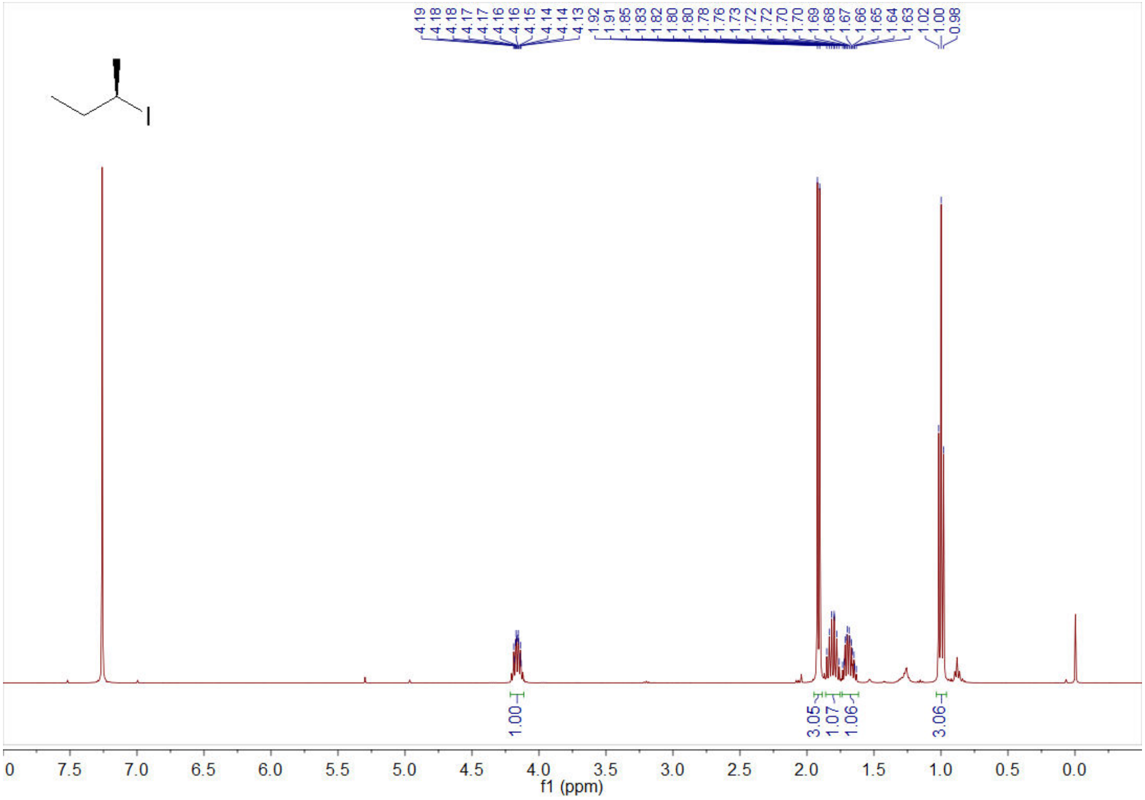

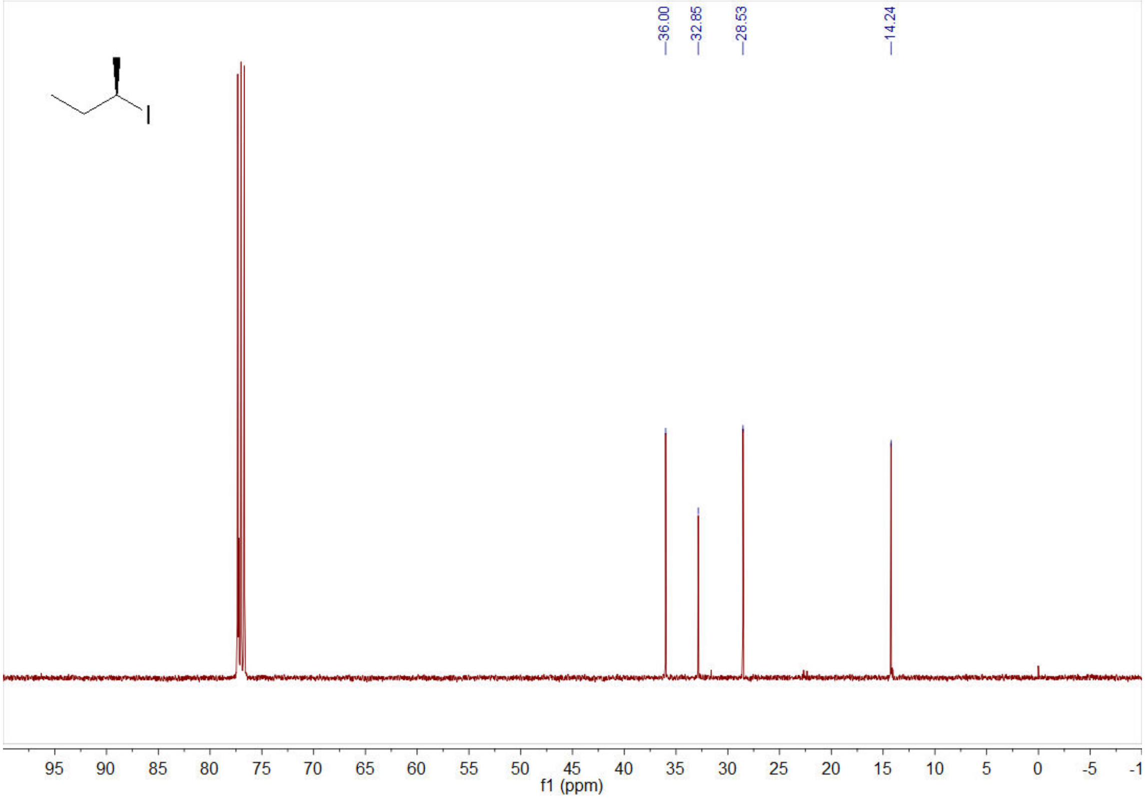

Supplementary Fig. 3. ^{1}H NMR (400 MHz, Chloroform-*d*) and ^{13}C NMR (101MHz, Chloroform-*d*) spectra of (R)-2-iodobutane.

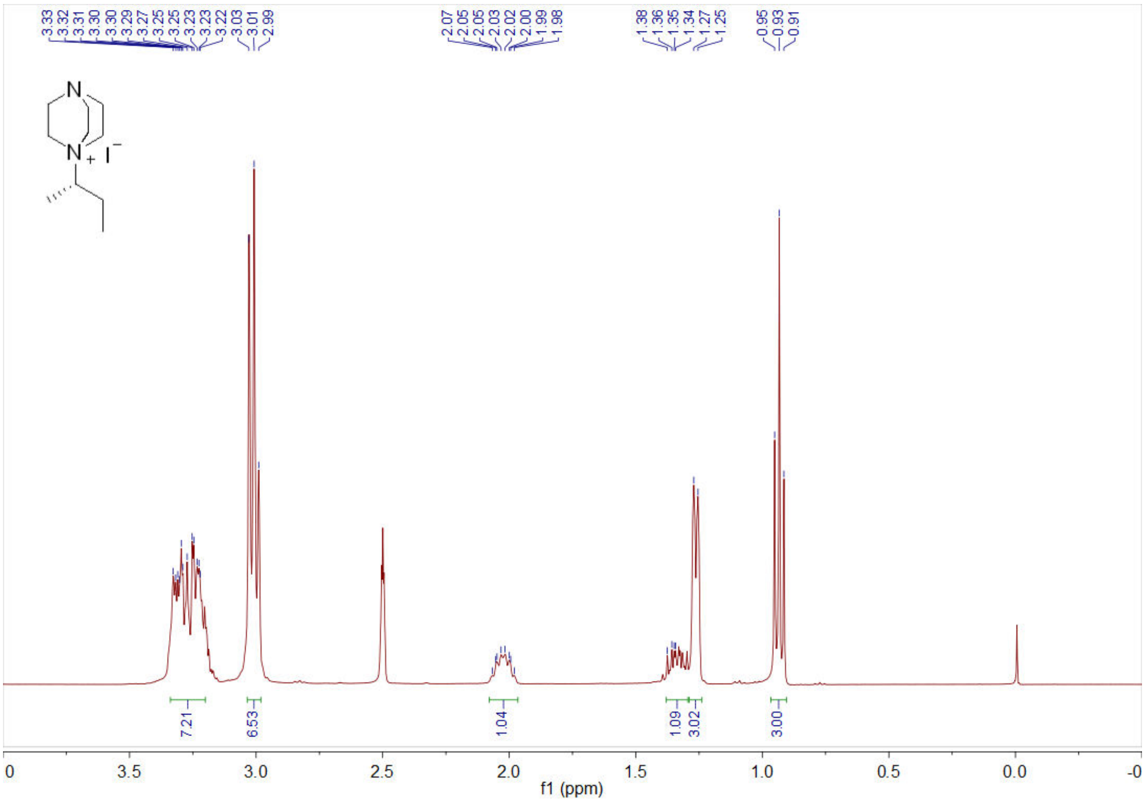

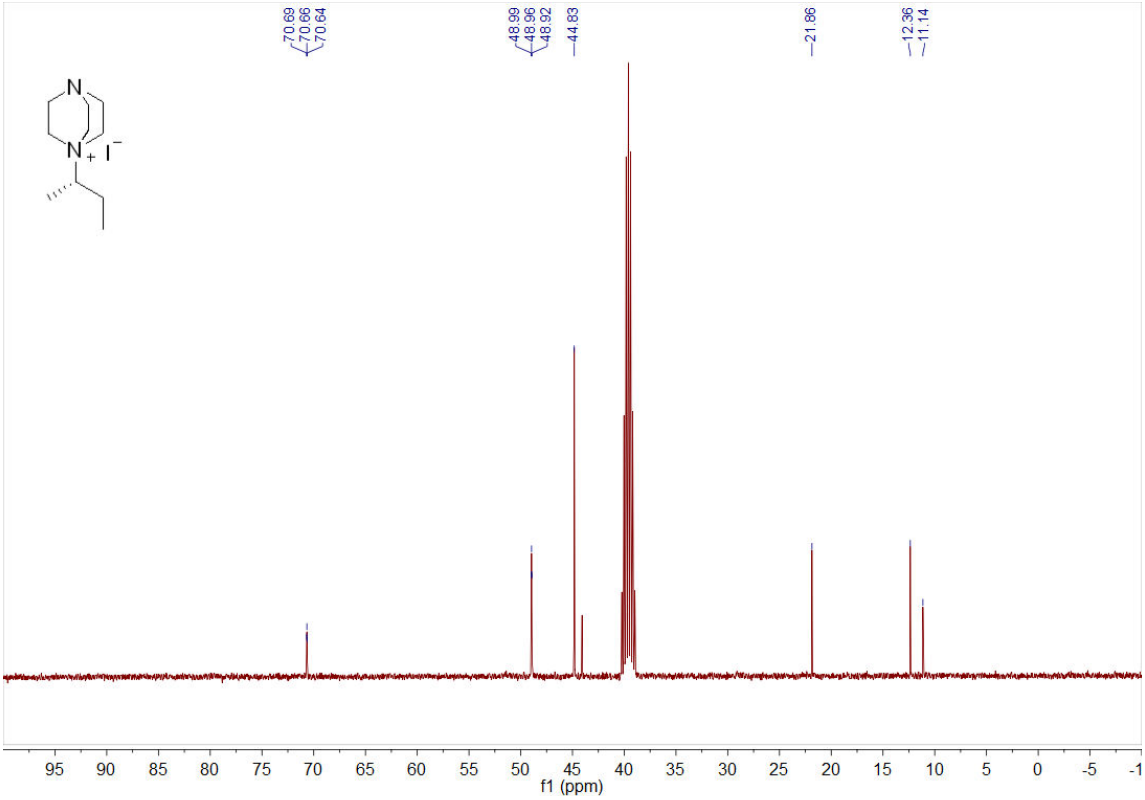

Supplementary Fig. 4. ^{1}H NMR (400 MHz, DMSO-d_6) and ^{13}C NMR (101MHz, DMSO-d_6) spectra of L_2.

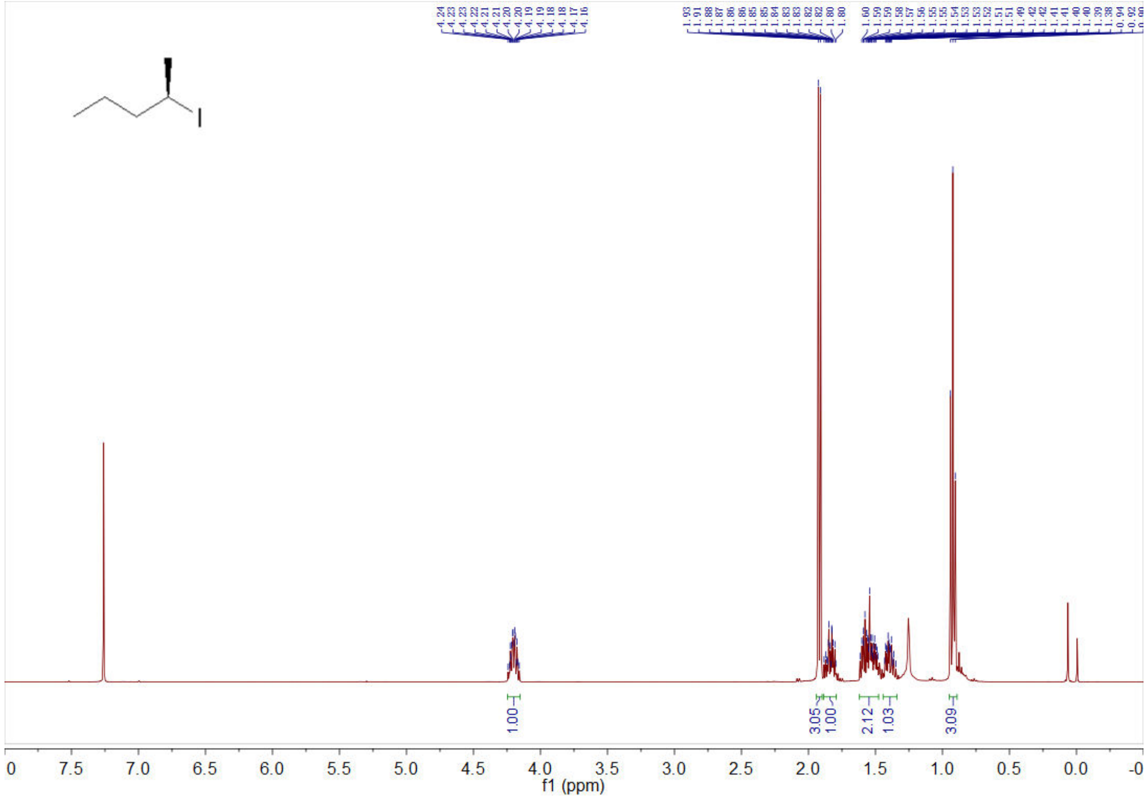

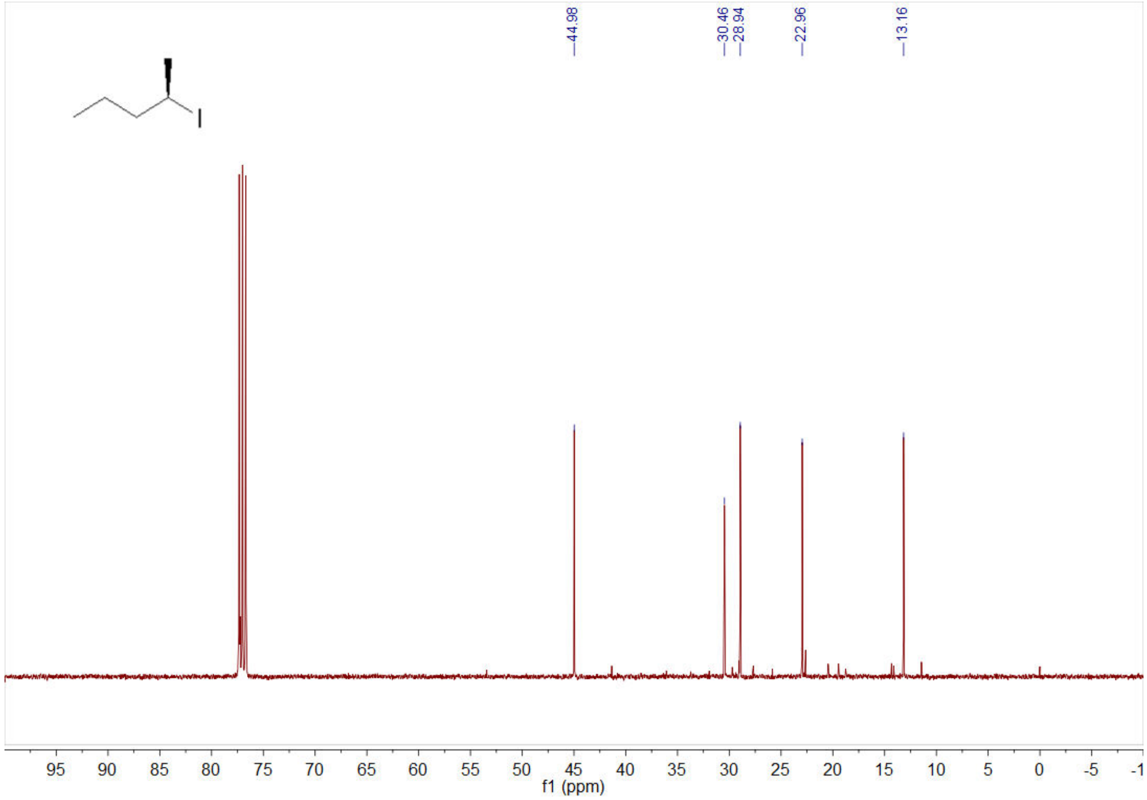

Supplementary Fig. 5. ^{1}H NMR (400 MHz, Chloroform-*d*) and ^{13}C NMR (101MHz, Chloroform-*d*) spectra of (R)-2-iodoheptane.

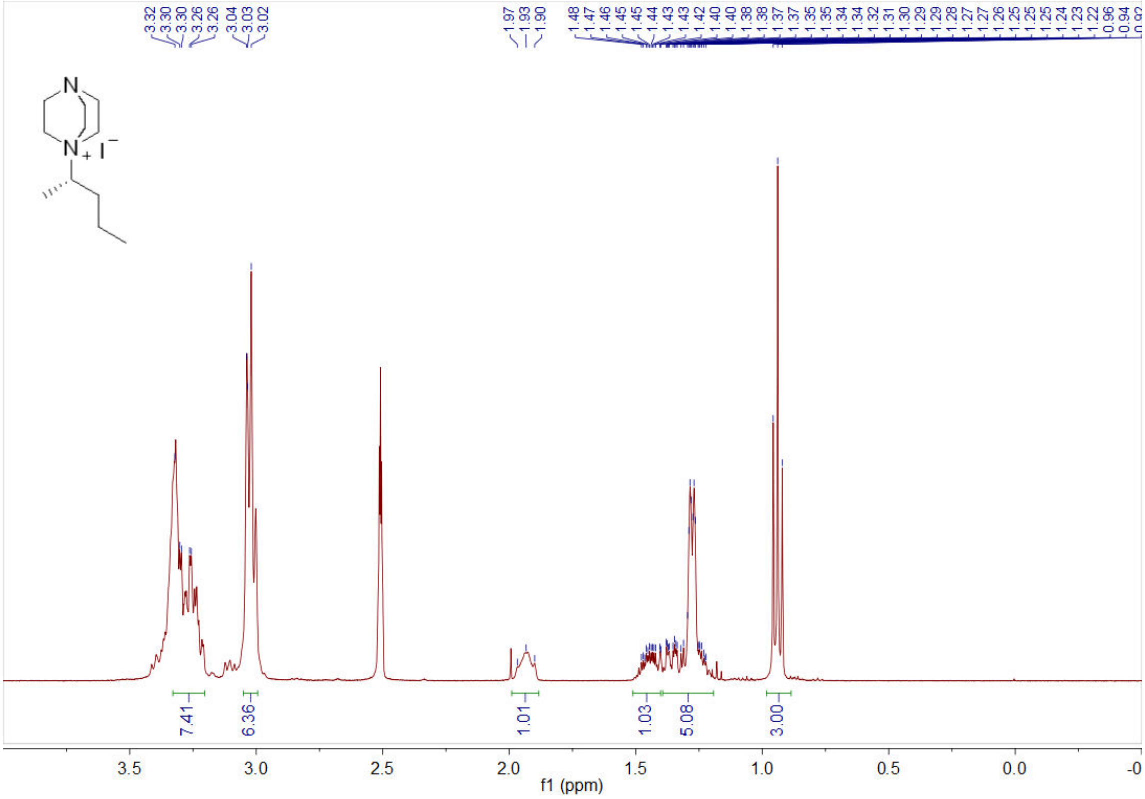

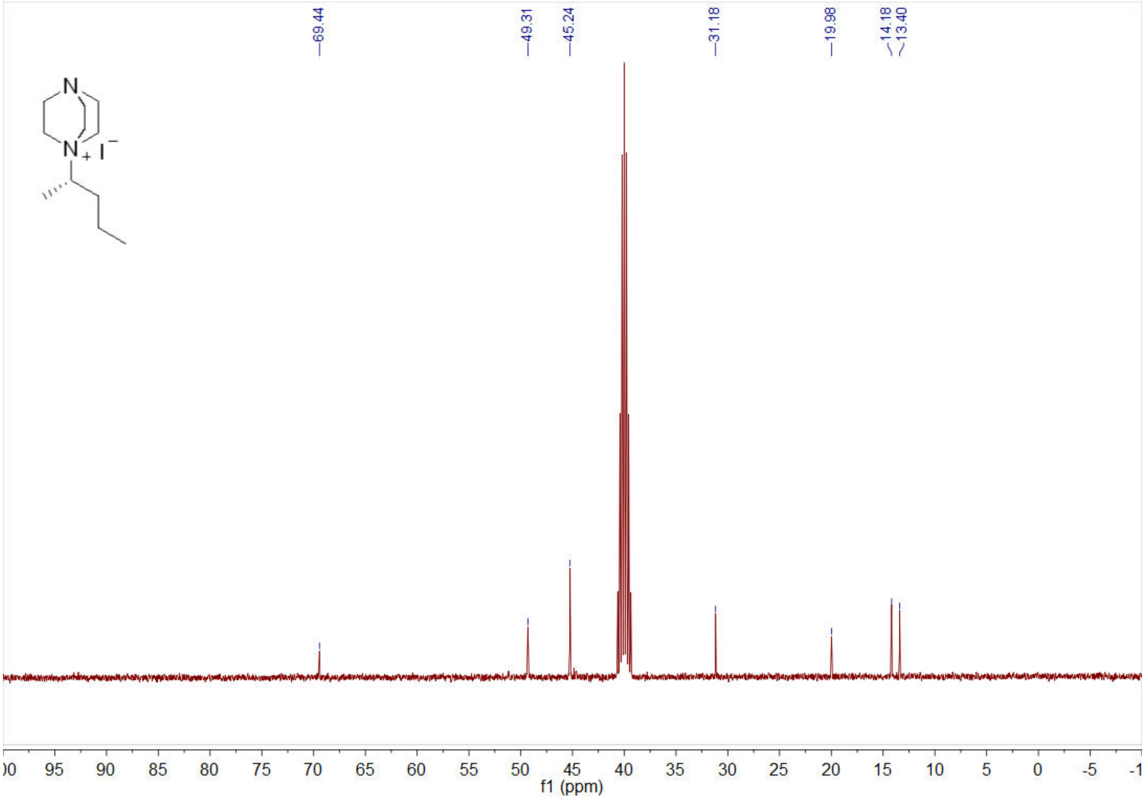

Supplementary Fig. 6. ^{1}H NMR (400 MHz, DMSO-d_6) and ^{13}C NMR (101MHz, DMSO-d_6) spectra of L_3.

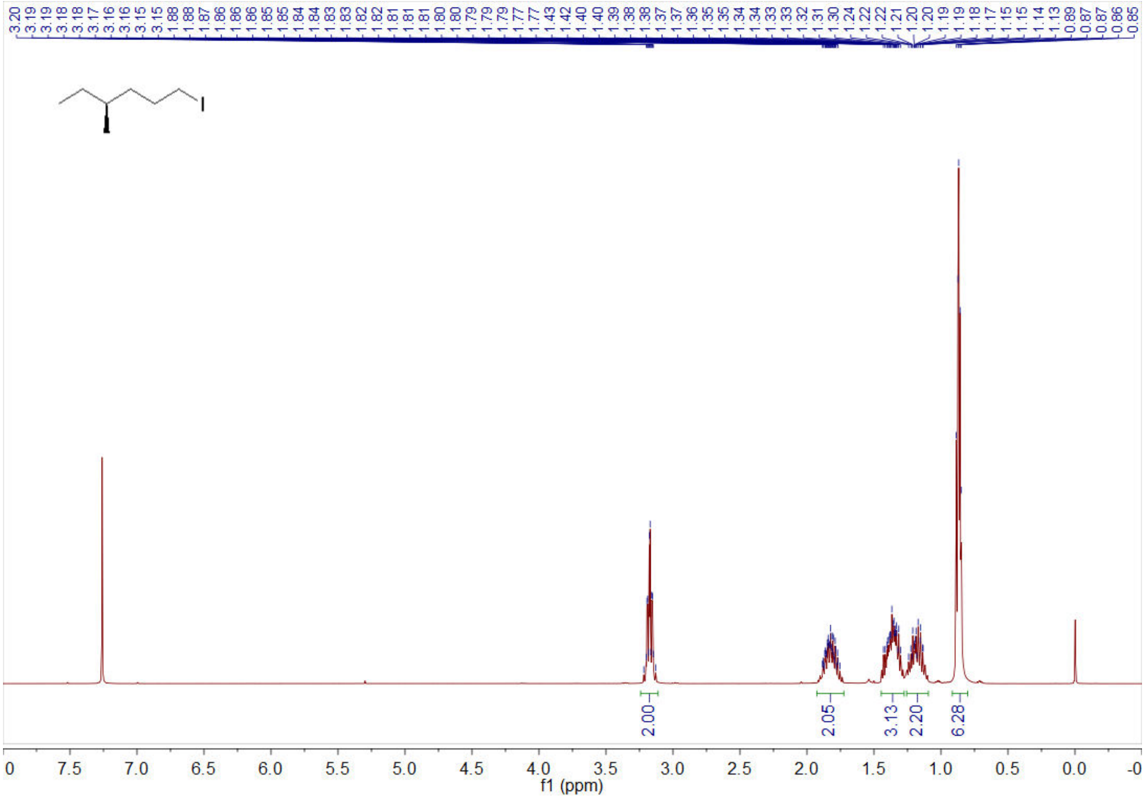

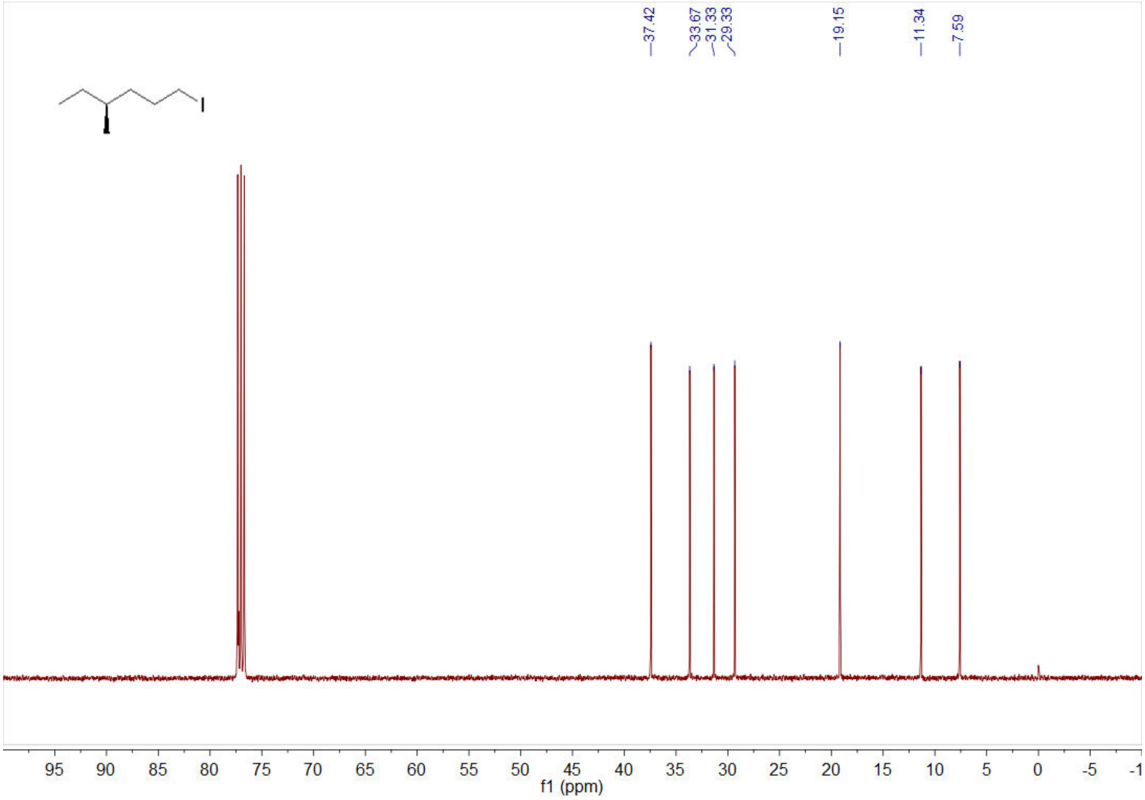

Supplementary Fig. 7. ^{1}H NMR (400 MHz, Chloroform-*d*) and ^{13}C NMR (101MHz, Chloroform-*d*) spectra of (S)-1-iodo-4-methylhexane.

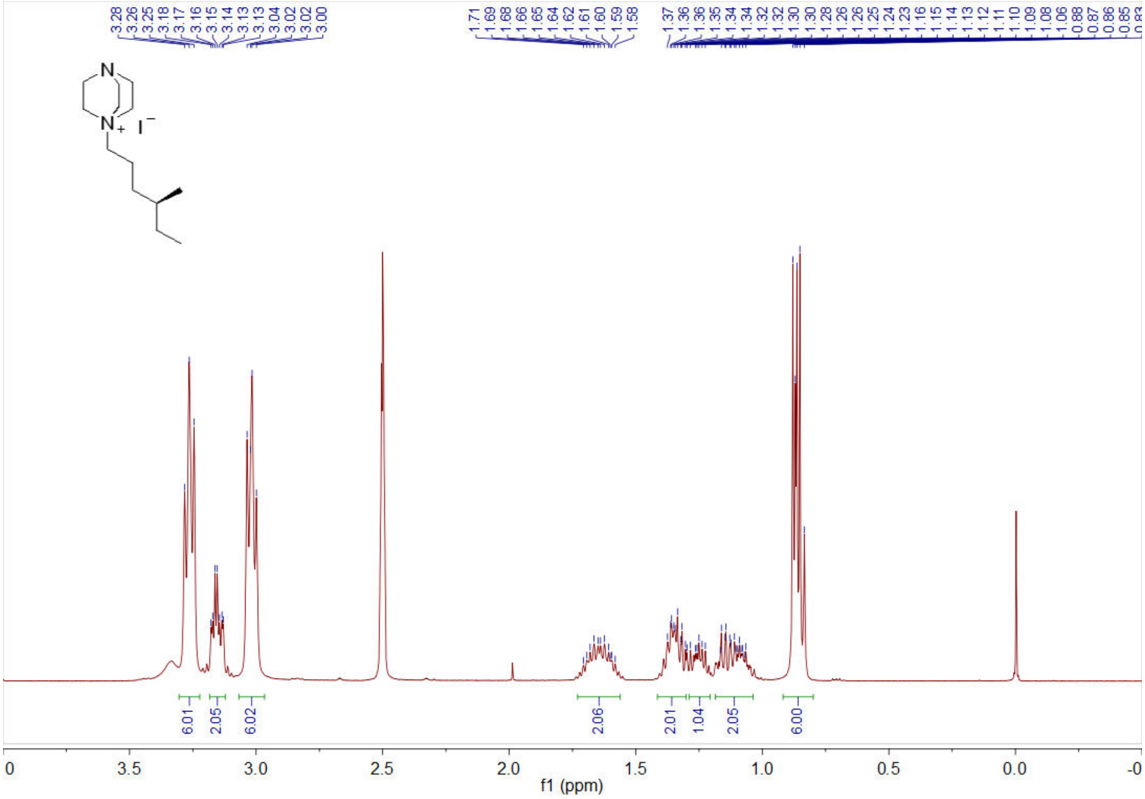

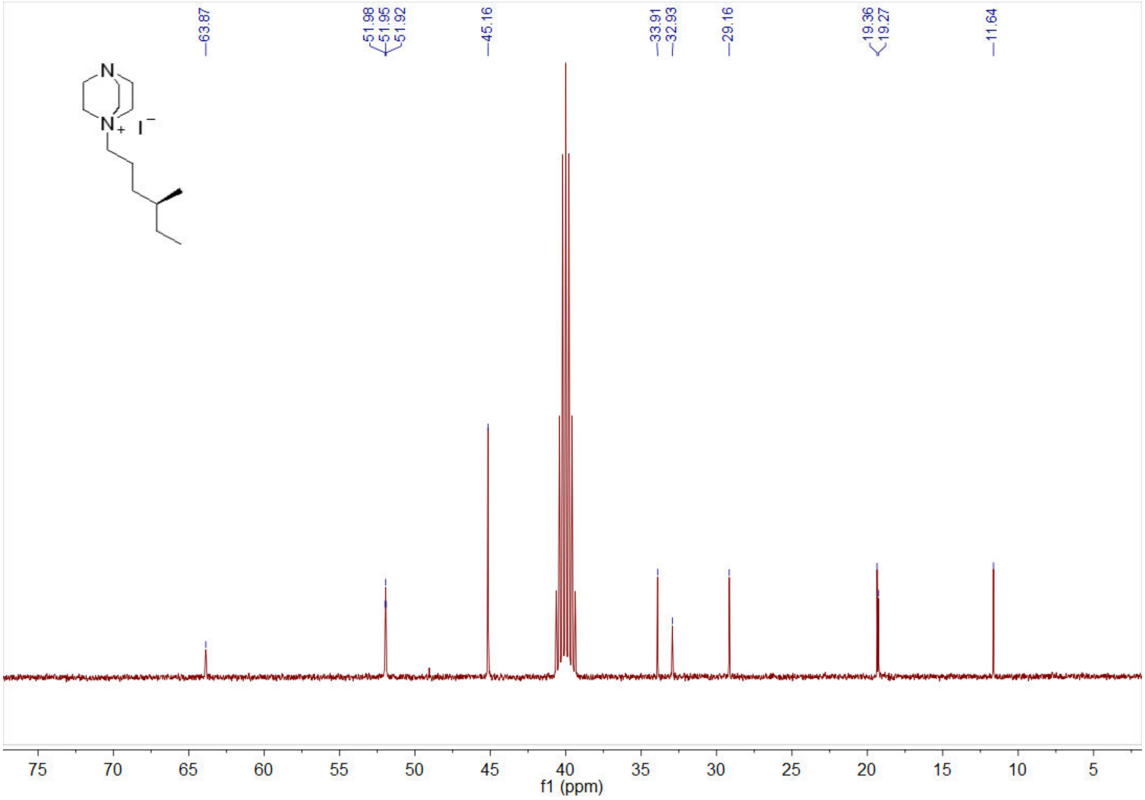

Supplementary Fig. 8. ^{1}H NMR (400 MHz, DMSO-d_6) and ^{13}C NMR (101MHz, DMSO-d_6) spectra of L_4.

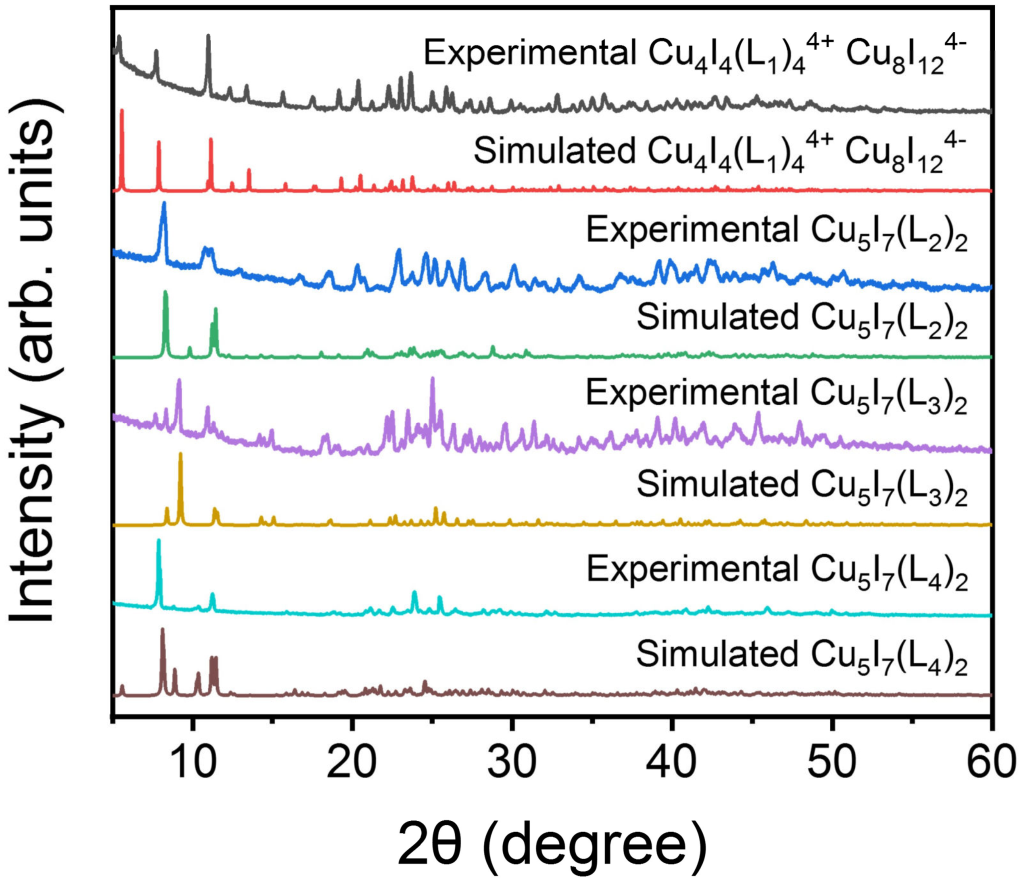

Supplementary Fig. 9. Experimental and simulated PXRD patterns of $Cu_4I_4(L_1)_4^{4+}$ $Cu_8I_{12}^{4-}$, $Cu_5I_7(L_2)_2$, $Cu_5I_7(L_3)_2$, and $Cu_5I_7(L_4)_2$. Source data are provided as a Source Data file.

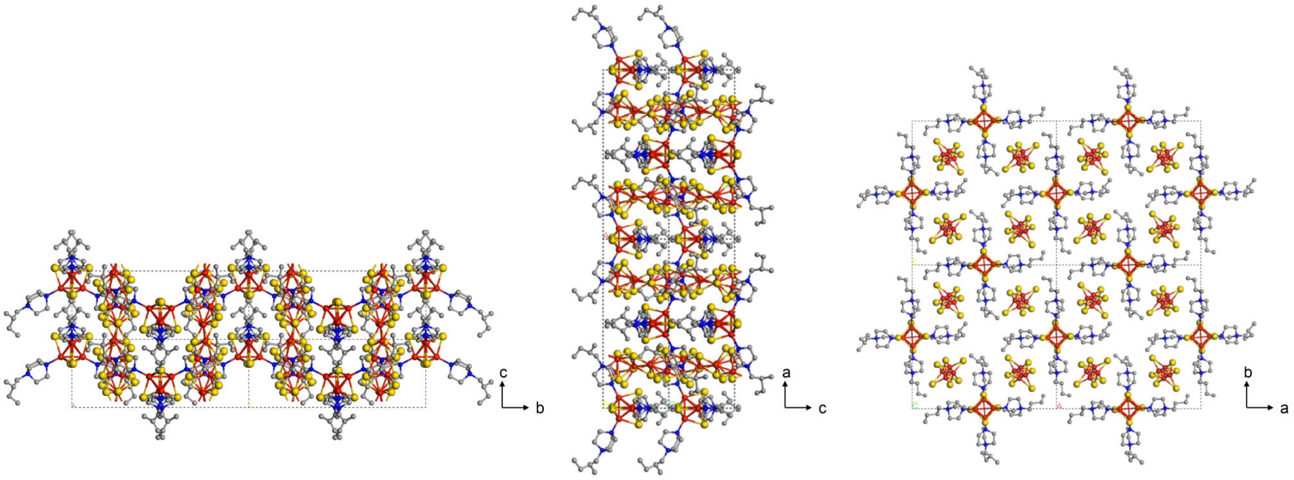

Supplementary Fig. 10. The spatial arrangement of $Cu_4I_4(L_1)_4^{4+}$ $Cu_8I_{12}^{4-}$ hybrid clusters in crystal lattices along a, b, c axes. Color scheme: Cu, red; I, yellow; N, blue; C, gray. The crystal structures are obtained by crystallography.

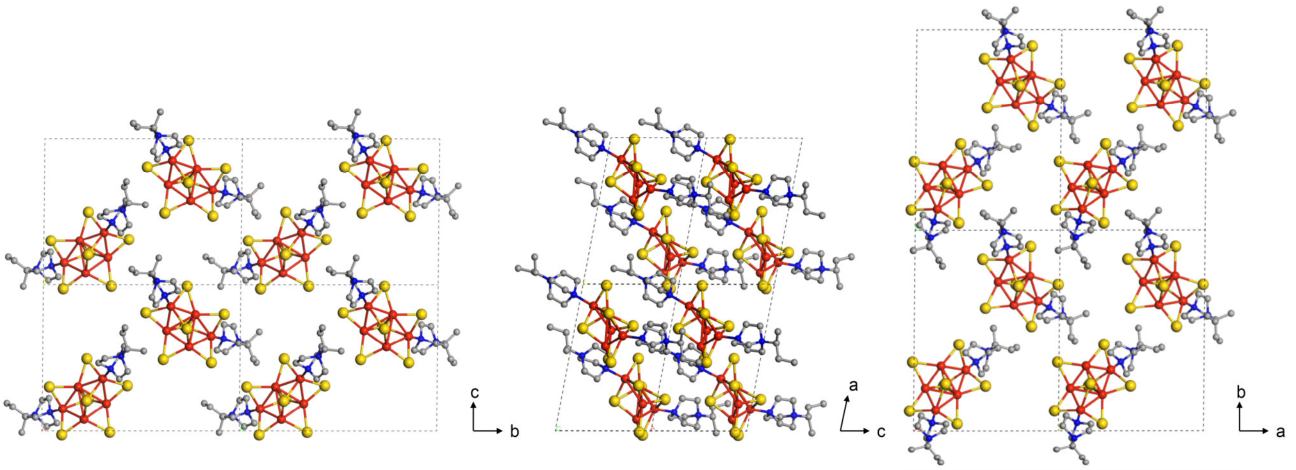

Supplementary Fig. 11. The spatial arrangement of $Cu_5I_7(L_2)_2$ hybrid clusters in crystal lattices along a, b, c axes. Color scheme: Cu, red; I, yellow; N, blue; C, gray. The crystal structures are obtained by crystallography.

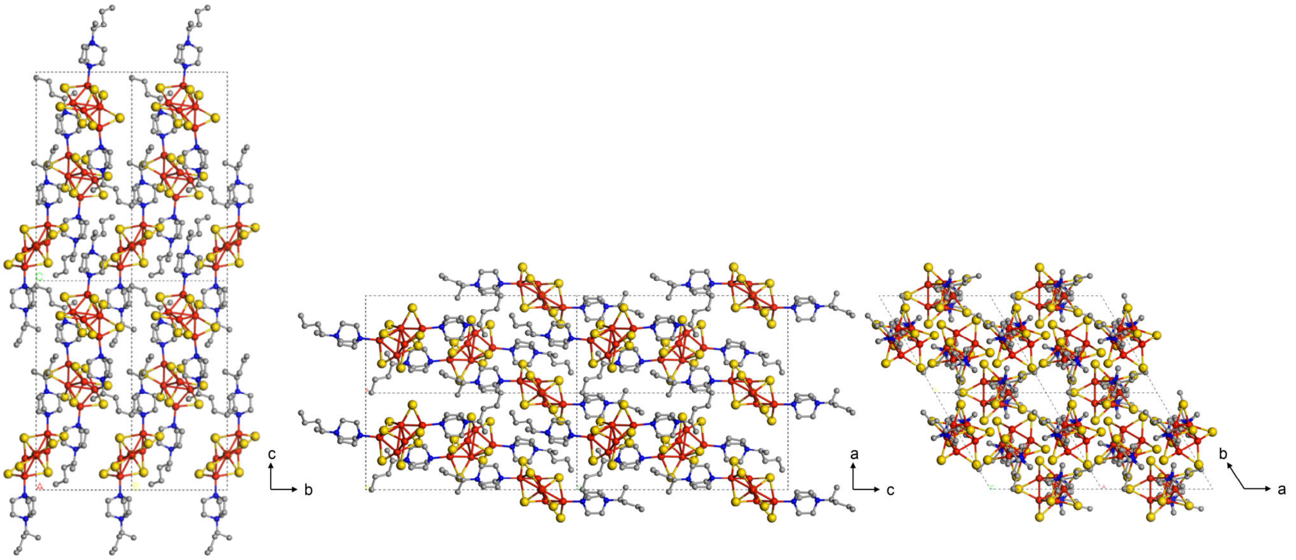

Supplementary Fig. 12. The spatial arrangement of $Cu_5I_7(L_3)_2$ hybrid clusters in crystal lattices along a, b, c axes. Color scheme: Cu, red; I, yellow; N, blue; C, gray. The crystal structures are obtained by crystallography.

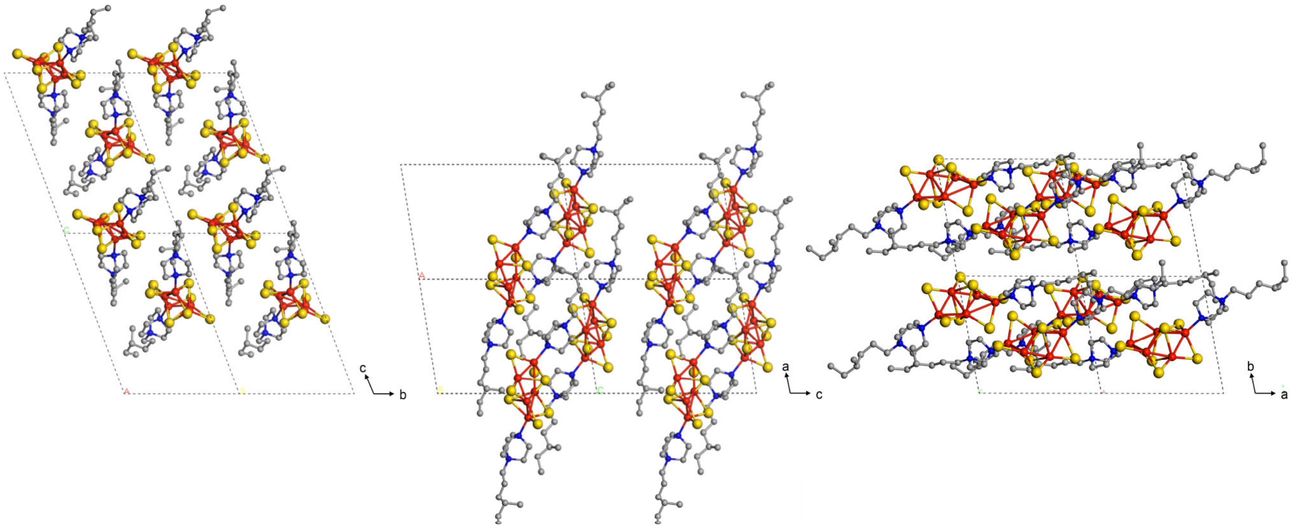

Supplementary Fig. 13. The spatial arrangement of $Cu_5I_7(L_4)_2$ hybrid clusters in crystal lattices along a, b, c axes. Color scheme: Cu, red; I, yellow; N, blue; C, gray. The crystal structures are obtained by crystallography.

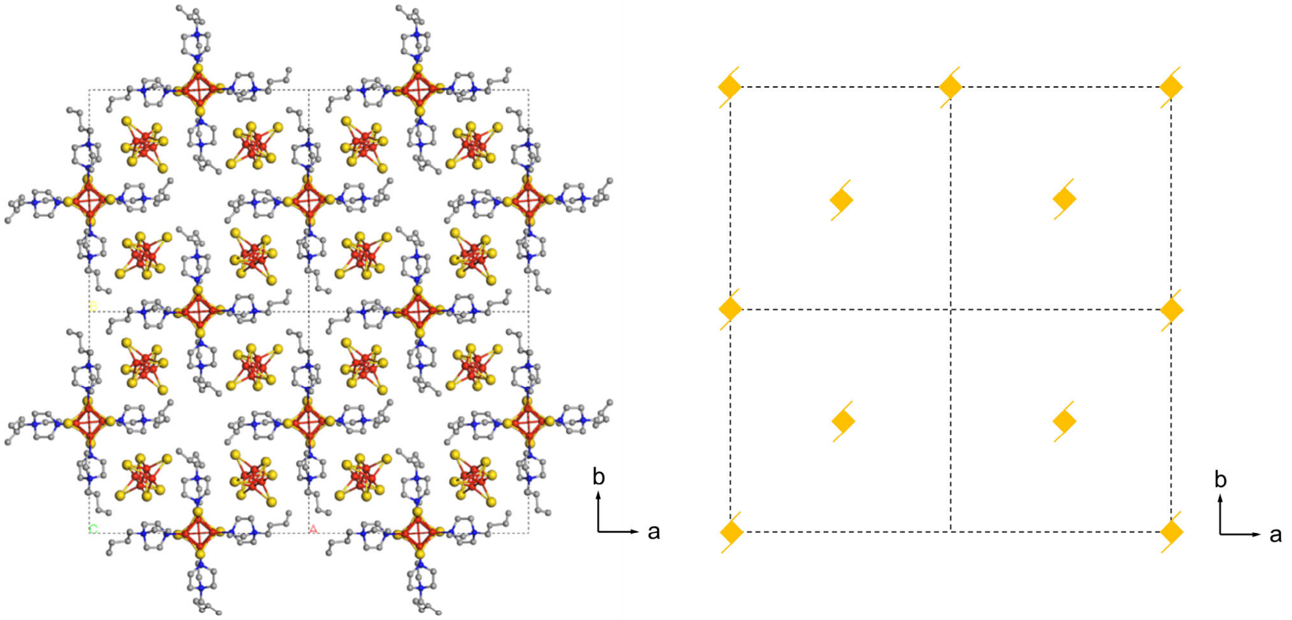

Supplementary Fig. 14. 4-Fold screw axis of $Cu_4I_4(L_1)_4^{4+}$ $Cu_8I_{12}^{4-}$ hybrid clusters in crystal lattices. The Cu-I inorganic chains were omitted in the helical arrangement structure of $Cu_4I_4(L_1)_4^{4+}$ $Cu_8I_{12}^{4-}$ for clarity. Color scheme: Cu, red; I, yellow; N, blue; C, gray. The crystal structures are obtained by crystallography.

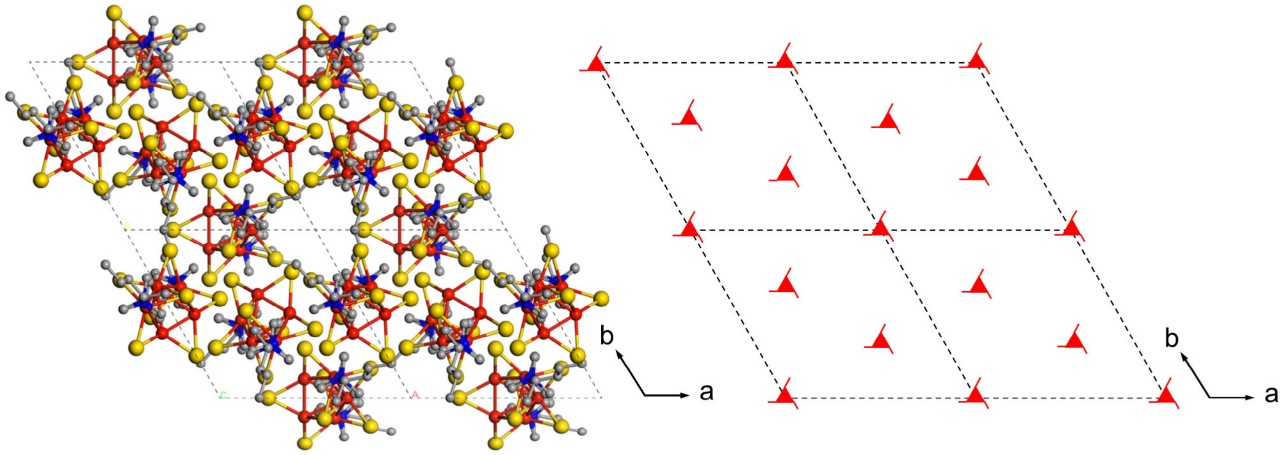

Supplementary Fig. 15. 3-Fold screw axis of $Cu_5I_7(L_3)_2$ hybrid clusters in crystal lattices. Color scheme: Cu, red; I, yellow; N, blue; C, gray. The crystal structures are obtained by crystallography.

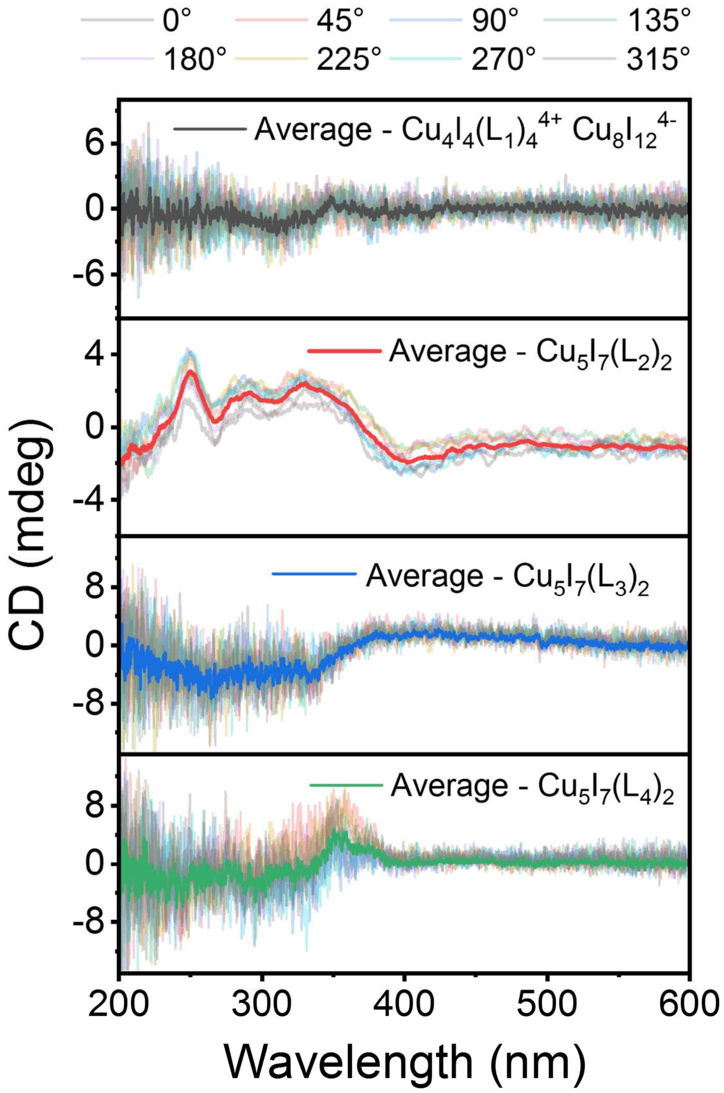

Supplementary Fig. 16. Angle-dependent CD spectra of $Cu_4I_4(L_1)_4^{4+}$ $Cu_8I_{12}^{4-}$, $Cu_5I_7(L_2)_2$, $Cu_5I_7(L_3)_2$ and $Cu_5I_7(L_4)_2$ fully ground single crystal powders. Source data are provided as a Source Data file.

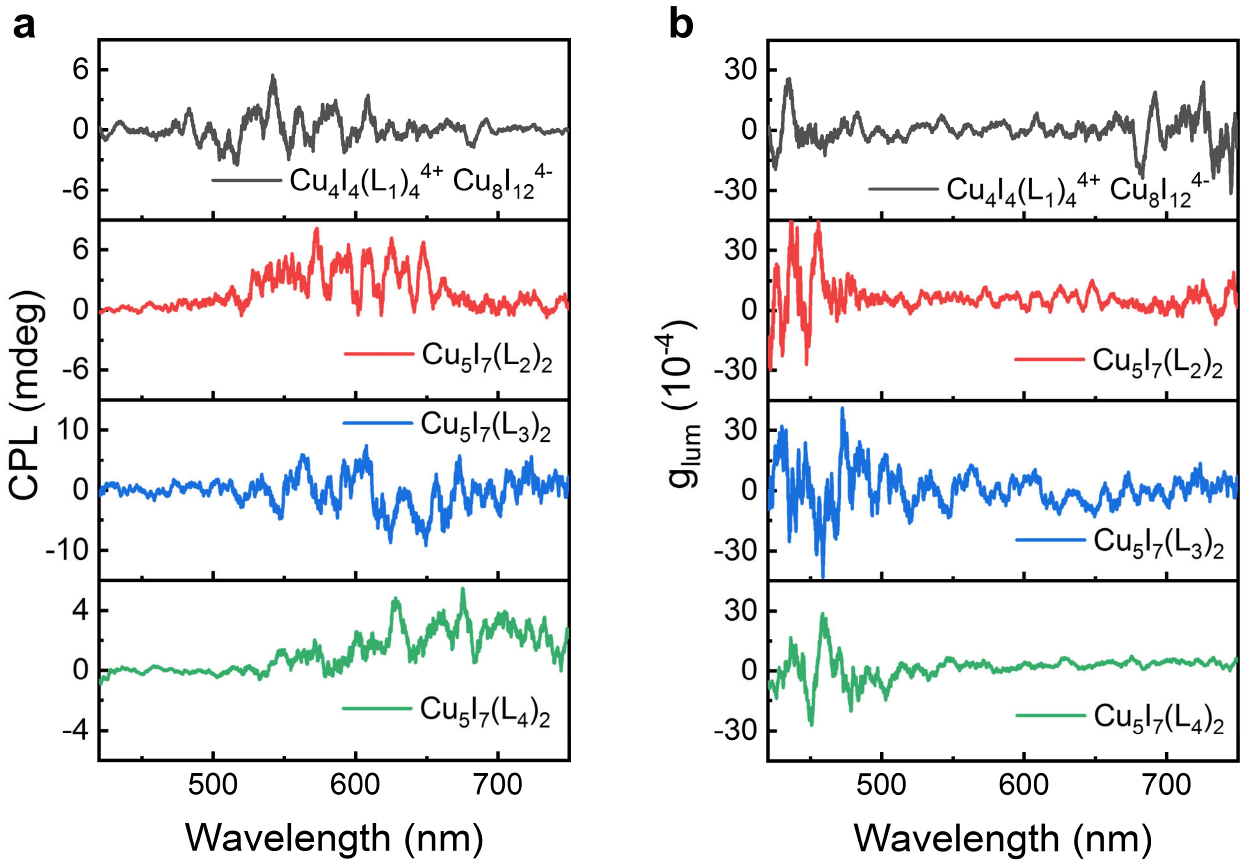

Supplementary Fig. 17. Circularly polarized luminescent properties of $Cu_4I_4(L_1)_4^{4+}$ $Cu_8I_{12}^{4-}$, $Cu_5I_7(L_2)_2$, $Cu_5I_7(L_3)_2$, and $Cu_5I_7(L_4)_2$ single crystal powders. a CPL spectra. **b** g_{lum} spectra. Source data are provided as a Source Data file.

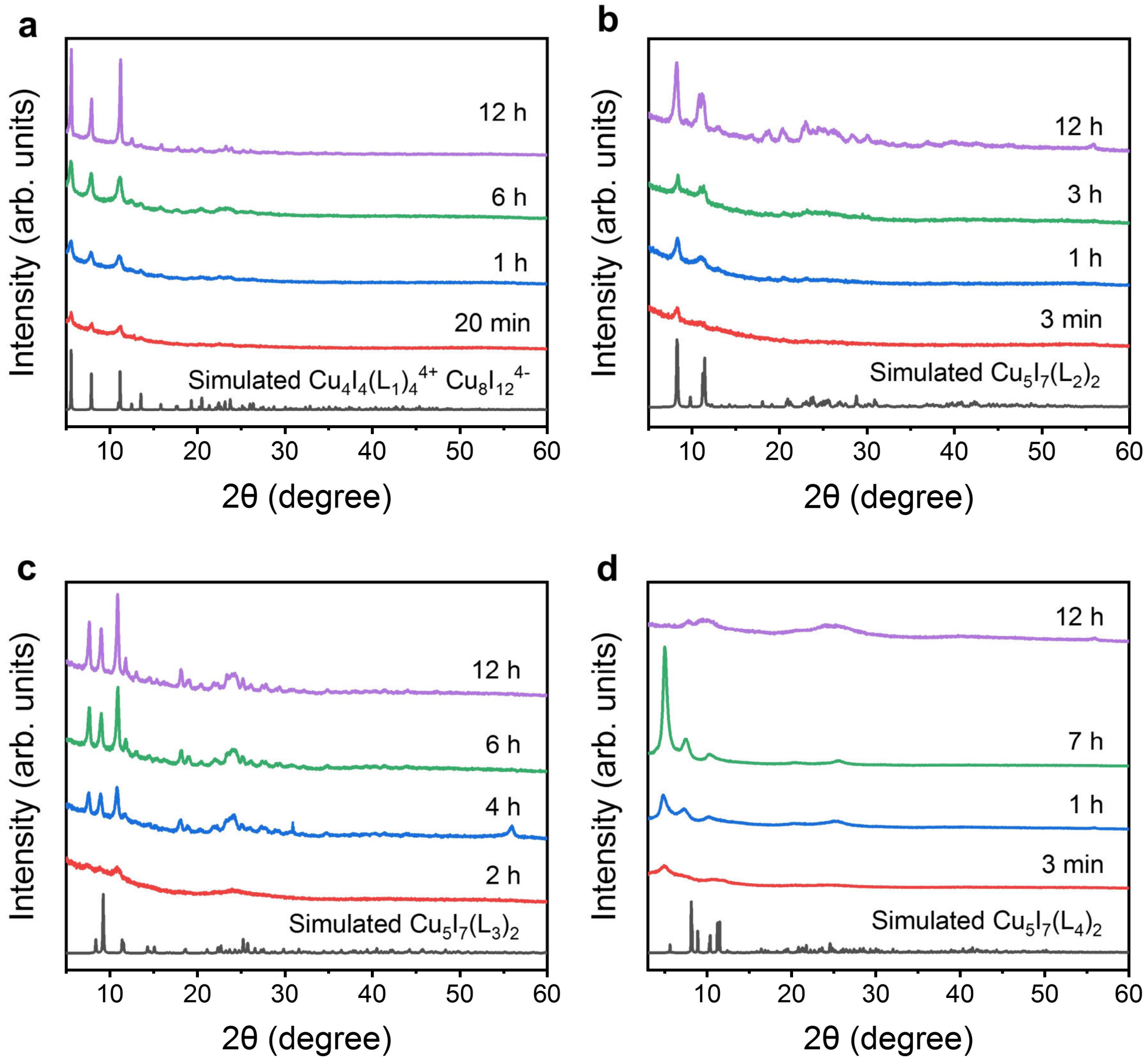

Supplementary Fig. 18. Crystalline phases of hierarchically structured polycrystals with different reaction times. a PXRD patterns of $Cu_4I_4(L_1)_4^{4+}$ $Cu_8I_{12}^{4-}$ polycrystals with different reaction time ($[L_1]$ = 6 mM). **b** PXRD patterns of $Cu_5I_7(L_2)_2$ polycrystals with different reaction time ($[L_2]$ = 20 mM). **c** PXRD patterns of $Cu_5I_7(L_3)_2$ polycrystals with different reaction time ($[L_3]$ = 25 mM). **d** PXRD patterns of $Cu_5I_7(L_4)_2$ polycrystals with different reaction time ($[L_4]$ = 20 mM). Source data are provided as a Source Data file.

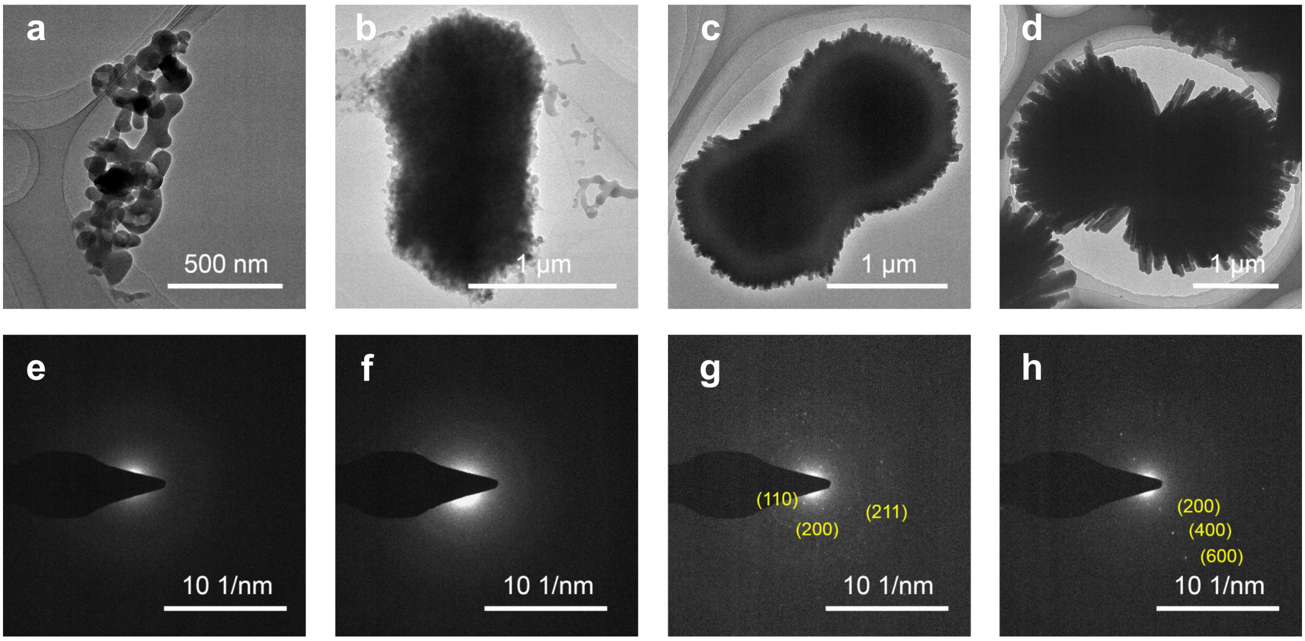

Supplementary Fig. 19. Morphology and Crystallinity changes of $Cu_4I_4(L_1)_4^{4+}$ $Cu_8I_{12}^{4-}$ polycrystals with different stages of crystallization. a-h TEM images (**a-d**) and SAEDs patterns (**e-h**) of different stages of crystallization for $Cu_4I_4(L_1)_4^{4+}$ $Cu_8I_{12}^{4-}$ polycrystals with the reaction time of 3 min (**a, e**), 20 min (**b, f**), 1 h (**c, g**), 6 h (**d, h**), respectively ([L_1] = 6 mM).

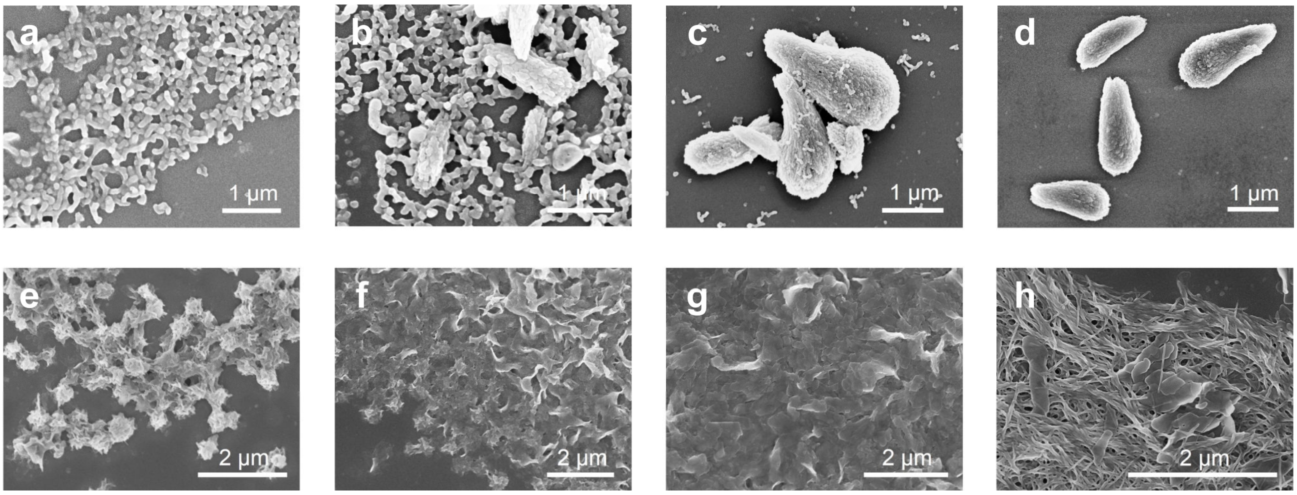

Supplementary Fig. 20. Morphology changes of $Cu_5I_7(L_2)_2$ and $Cu_5I_7(L_4)_2$ polycrystals with different stages of crystallization. a-d SEM images of different stages of crystallization for $Cu_5I_7(L_2)_2$ polycrystals with reaction time of 3 min (**a**), 2 h (**b**), 3 h (**c**), 6 h (**d**), respectively ($[L_2]$ = 20 mM). **e-h** SEM images of different stages of crystallization for $Cu_5I_7(L_4)_2$ polycrystals with reaction time as 3 min (**e**), 1 h (**f**), 7 h (**g**), 12 h (**h**), respectively ($[L_4]$ = 20 mM).

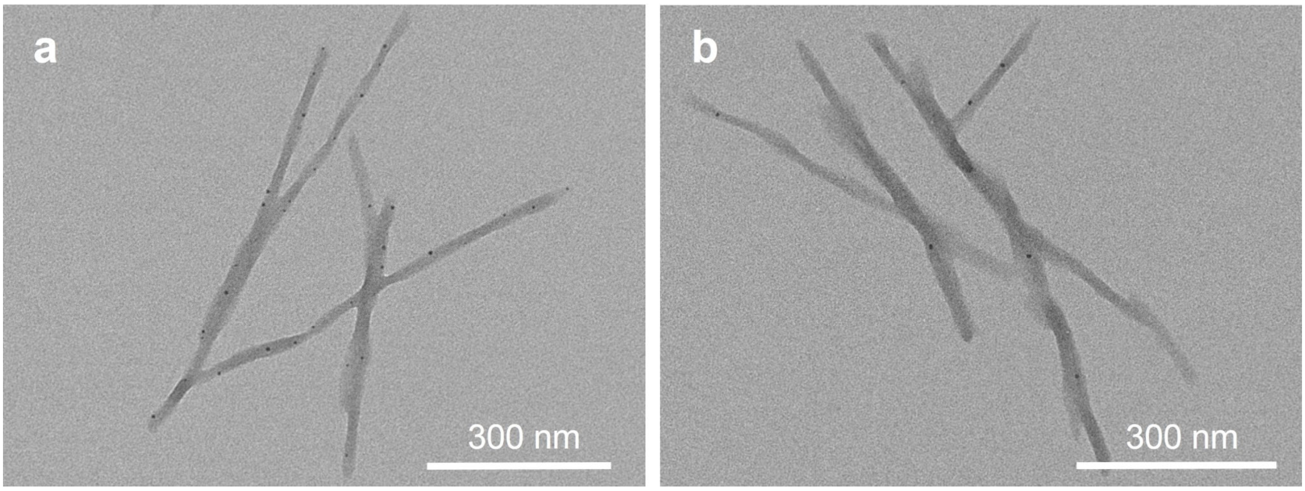

Supplementary Fig. 21. TEM images of $Cu_5I_7(L_4)_2$ helical nanobelts (**a-b**).

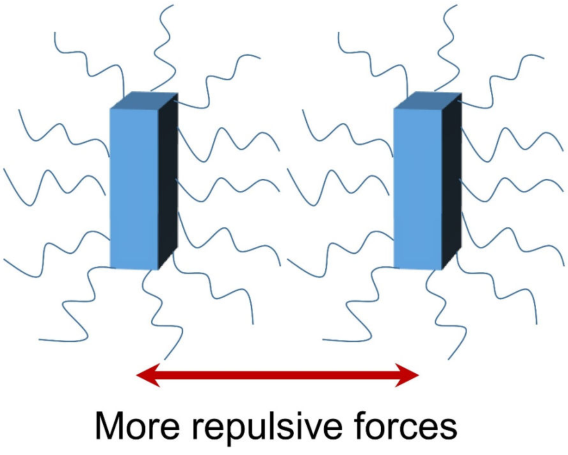

Supplementary Fig. 22. Schematic diagram of repulsive forces between assembly units caused by long (left) and short (right) chains of ligands absorbed on the surfaces, respectively. Blue cuboids represent the assembly units, and the wavy lines attached to the surfaces of cuboids represent ligands.

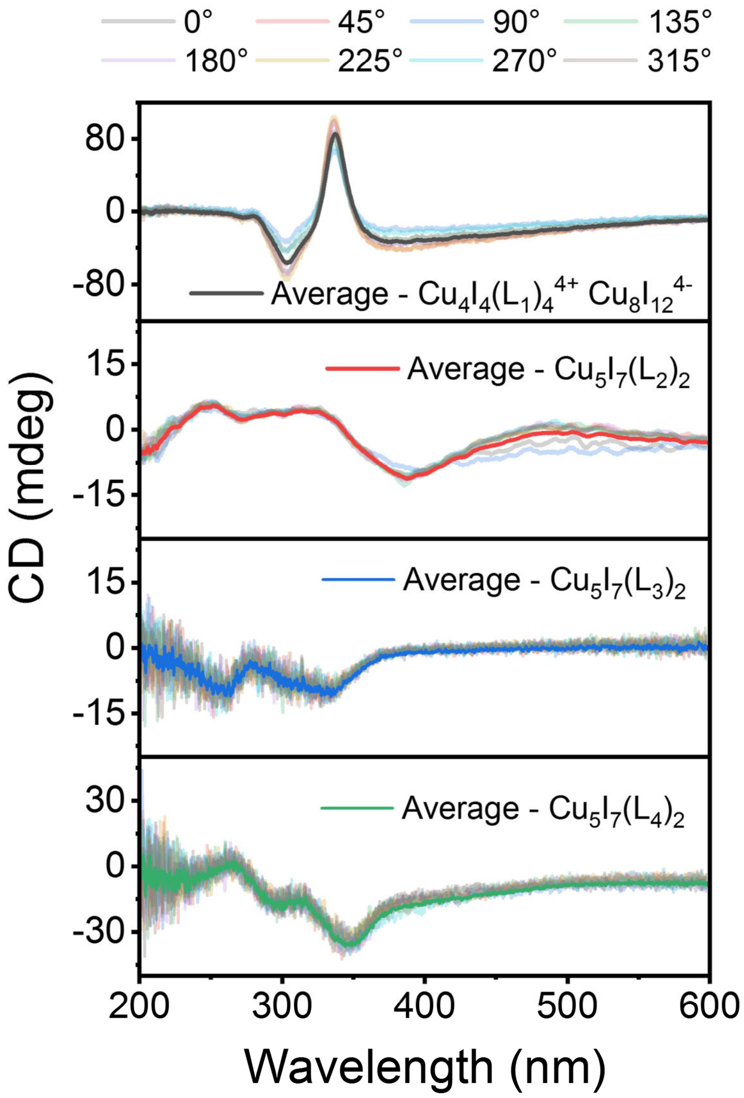

Supplementary Fig. 23. Angle-dependent CD spectra of $Cu_4I_4(L_1)_4^{4+}$ $Cu_8I_{12}^{4-}$, $Cu_5I_7(L_2)_2$, $Cu_5I_7(L_3)_2$, and $Cu_5I_7(L_4)_2$ hierarchically structured polycrystals with the concentrations of L_1-L_4 as 6 mM, 20 mM, 25 mM, 20 mM, respectively. Source data are provided as a Source Data file.

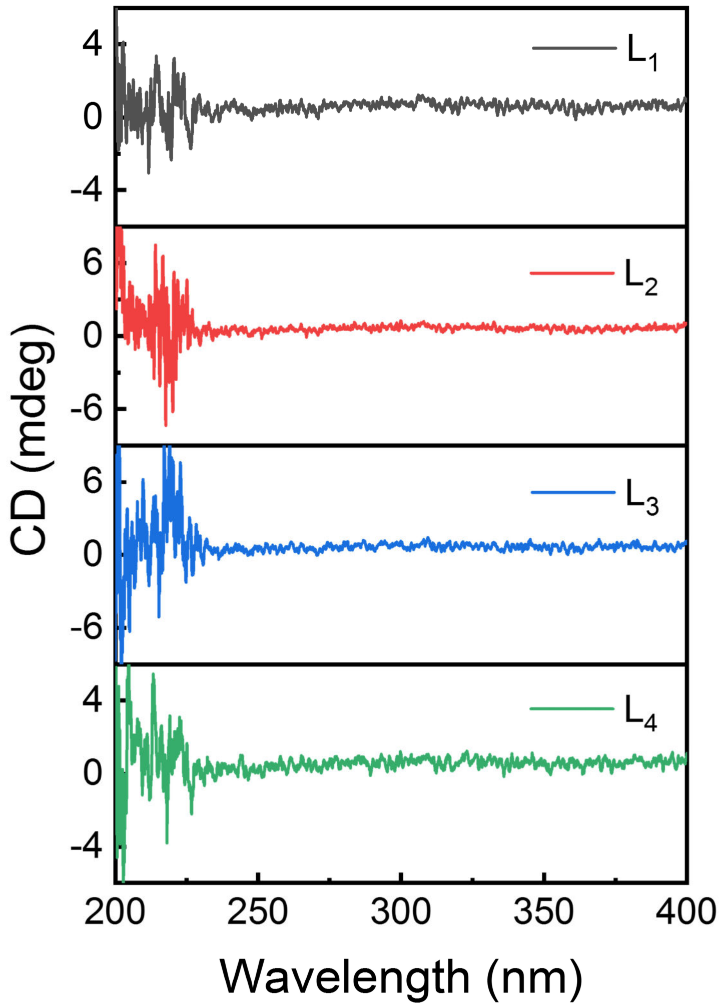

Supplementary Fig. 24. CD spectra of ligands L_1, L_2, L_3, and L_4. Source data are provided as a Source Data file.

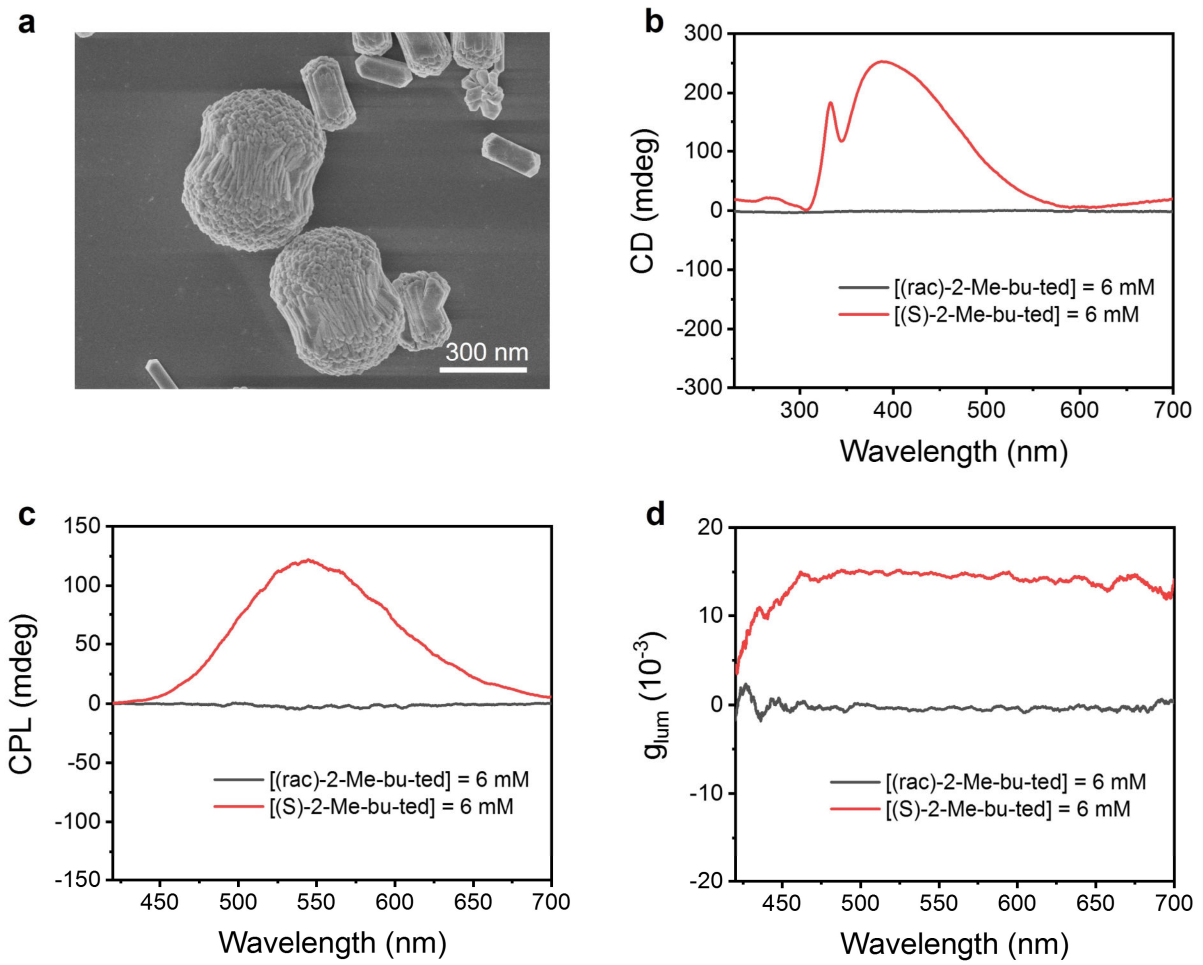

Supplementary Fig. 25. Comparison of $Cu_4I_4(L_1)_4^{4+}$ $Cu_8I_{12}^{4-}$ hierarchically structured polycrystals synthesized by racemic and chiral ligands. a SEM images of (rac)-$Cu_4I_4(L_1)_4^{4+}$ $Cu_8I_{12}^{4-}$ hierarchically structured polycrystals synthesized by racemic L_1 ligands. **b-d** CD (**b**), CPL (**c**), and g_{lum} (**d**) spectra of hierarchically structured polycrystals synthesized by racemic and chiral L_1 ligands, respectively. Other conditions: [racemic L_1] = [L_1] = 6 mM. Source data are provided as a Source Data file.

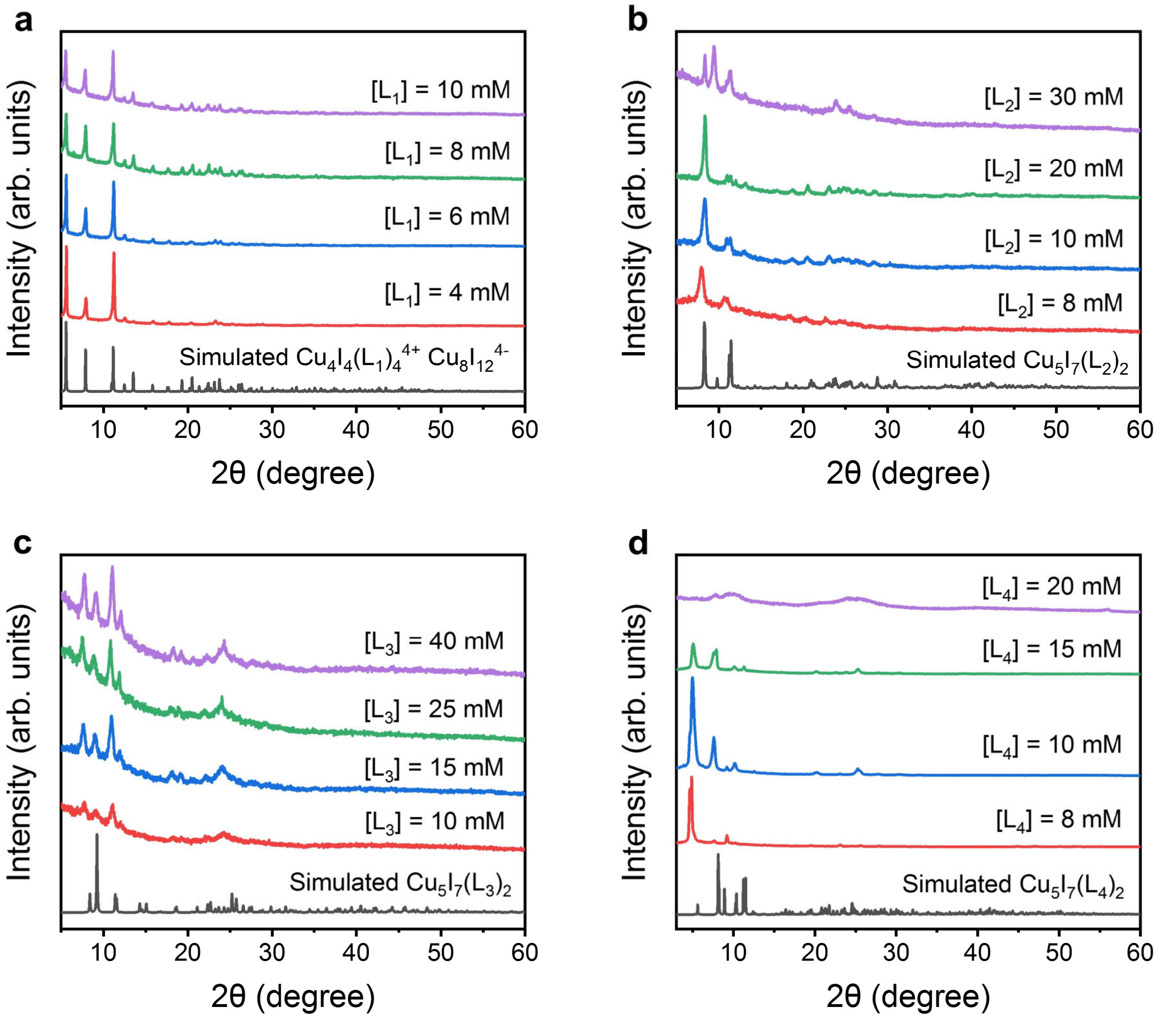

Supplementary Fig. 26. Crystalline phases of hierarchically structured polycrystals with different concentrations of ligands. a-d PXRD patterns of $Cu_4I_4(L_1)_4^{4+}$ $Cu_8I_{12}^{4-}$ (**a**), $Cu_5I_7(L_2)_2$ (**b**), $Cu_5I_7(L_3)_2$ (**c**), and $Cu_5I_7(L_4)_2$ (**d**) polycrystals with different concentrations of ligands. Source data are provided as a Source Data file.

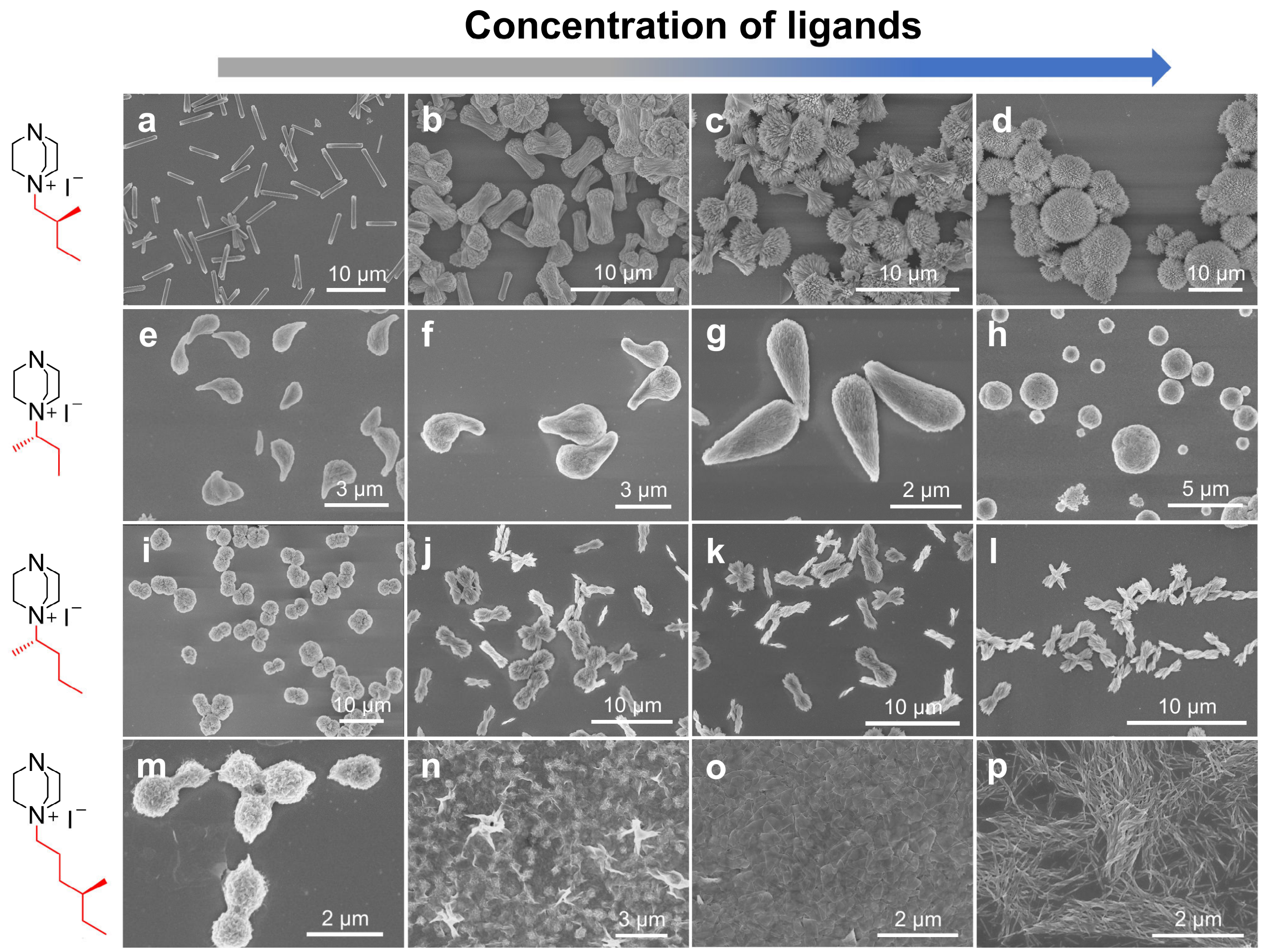

Supplementary Fig. 27. Large-scale SEM images of hierarchically structured polycrystals with different concentrations of ligands. a-d SEM images of $Cu_4I_4(L_1)_4^{4+}$ $Cu_8I_{12}^{4-}$ polycrystals with the concentrations of L_1 as 4 mM (**a**), 5 mM (**b**), 6 mM (**c**), 10 mM (**d**), respectively. **e-h** SEM images of $Cu_5I_7(L_2)_2$ polycrystals with concentrations of L_2 as 5 mM (**e**), 10 mM (**f**), 20 mM (**g**), 30 mM (**h**), respectively. **i-l** SEM images of $Cu_5I_7(L_3)_2$ polycrystals with concentrations of L_3 as 10 mM (**i**), 20 mM (**j**), 25 mM (**k**), 40 mM (**l**), respectively. **m-p** SEM images of $Cu_5I_7(L_4)_2$ polycrystals with concentration of L_4 as 3 mM (**m**), 5 mM (**n**), 10 mM (**o**), 20 mM (**p**), respectively.

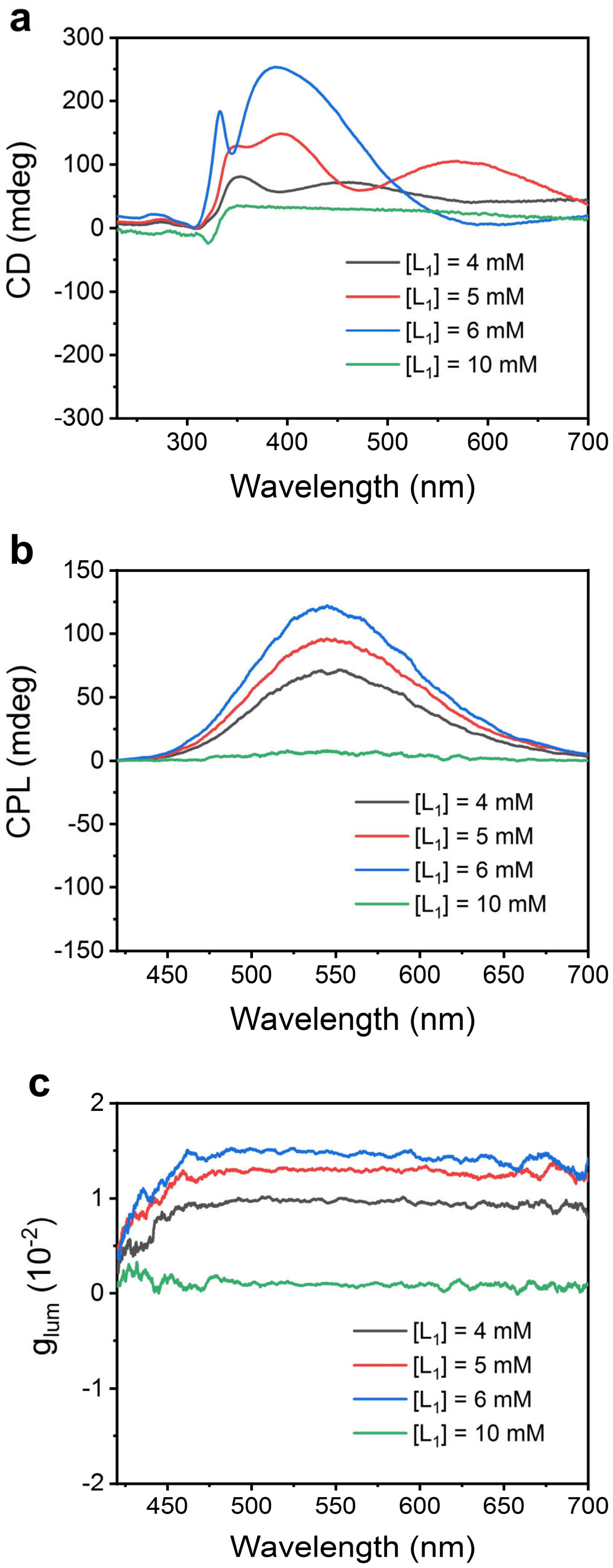

Supplementary Fig. 28. Chiroptical properties of $Cu_4I_4(L_1)_4^{4+}$ $Cu_8I_{12}^{4-}$ hierarchically chiral polycrystals with different concentrations of L_1. a CD spectra. **b** CPL spectra. **c** g_{lum} spectra. Source data are provided as a Source Data file.

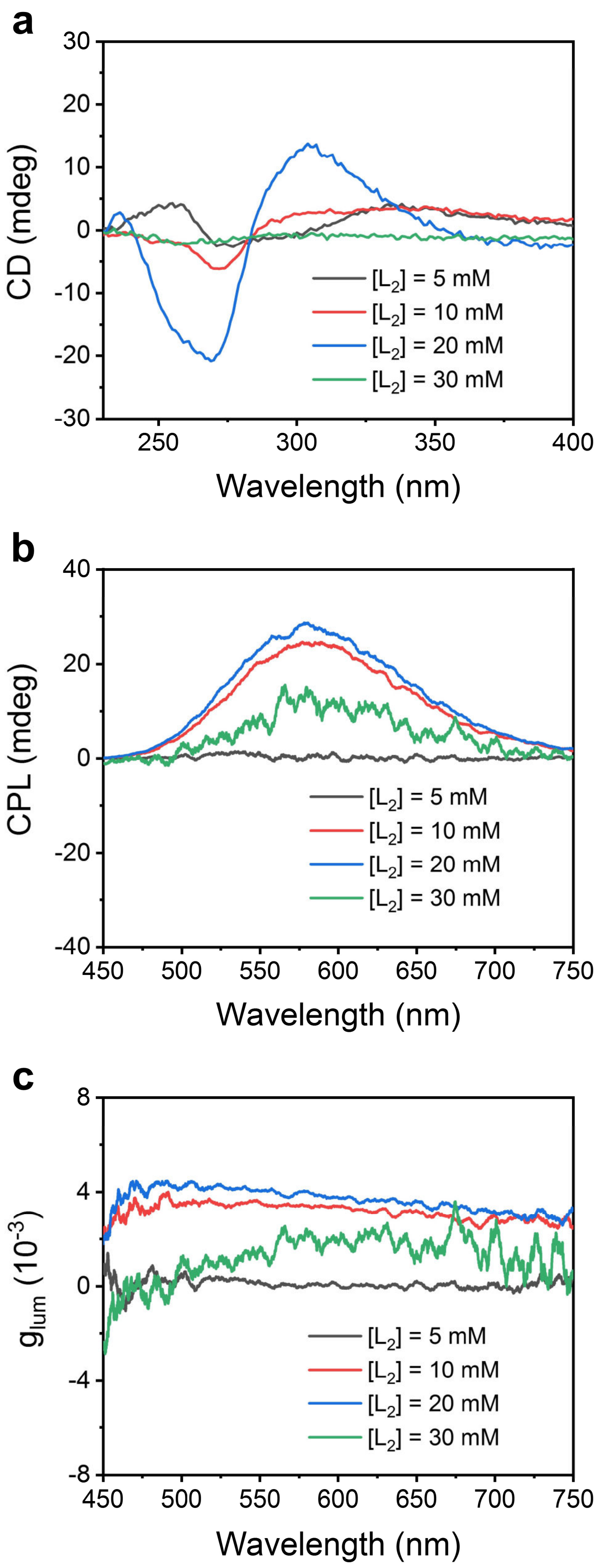

Supplementary Fig. 29. Chiroptical properties of $Cu_5I_7(L_2)_2$ hierarchically chiral polycrystals with different concentrations of L_2. a CD spectra. **b** CPL spectra. **c** g_{lum} spectra. Source data are provided as a Source Data file.

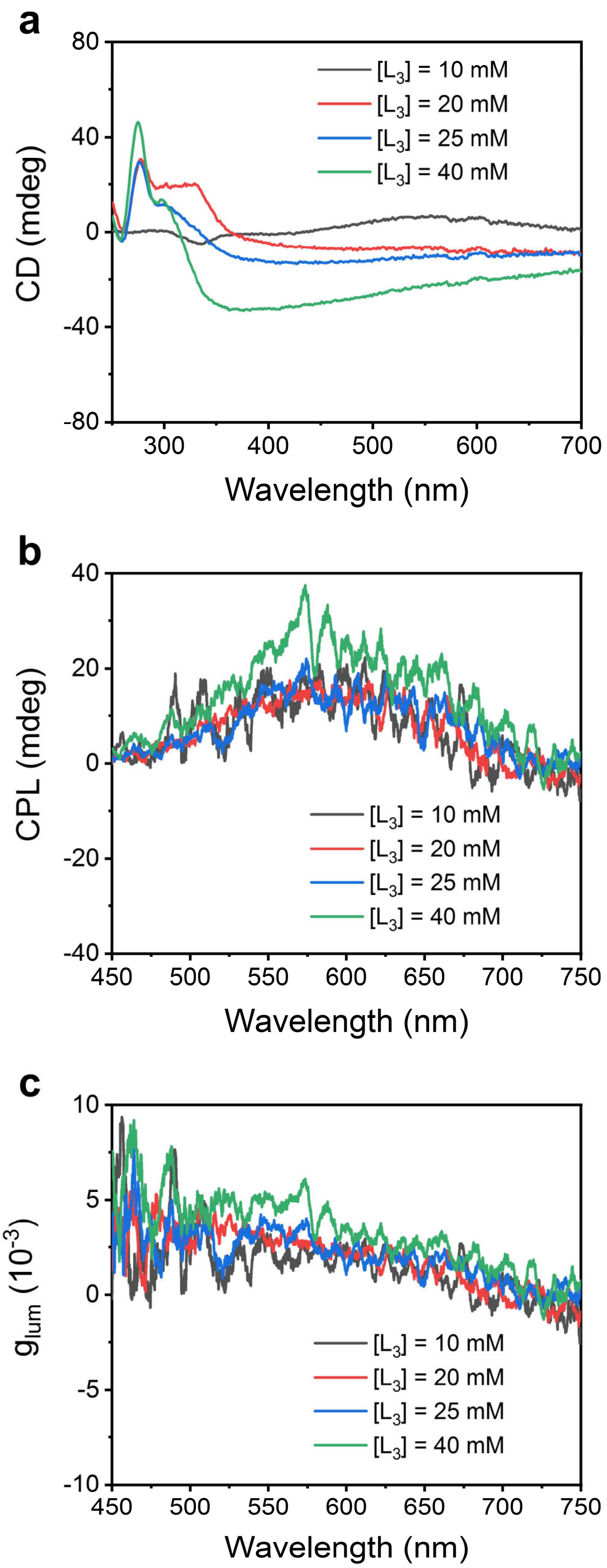

Supplementary Fig. 30. Chiroptical properties of $Cu_5I_7(L_3)_2$ hierarchically chiral polycrystals with different concentrations of L_3. a CD spectra. **b** CPL spectra. **c** g_{lum} spectra. Source data are provided as a Source Data file.

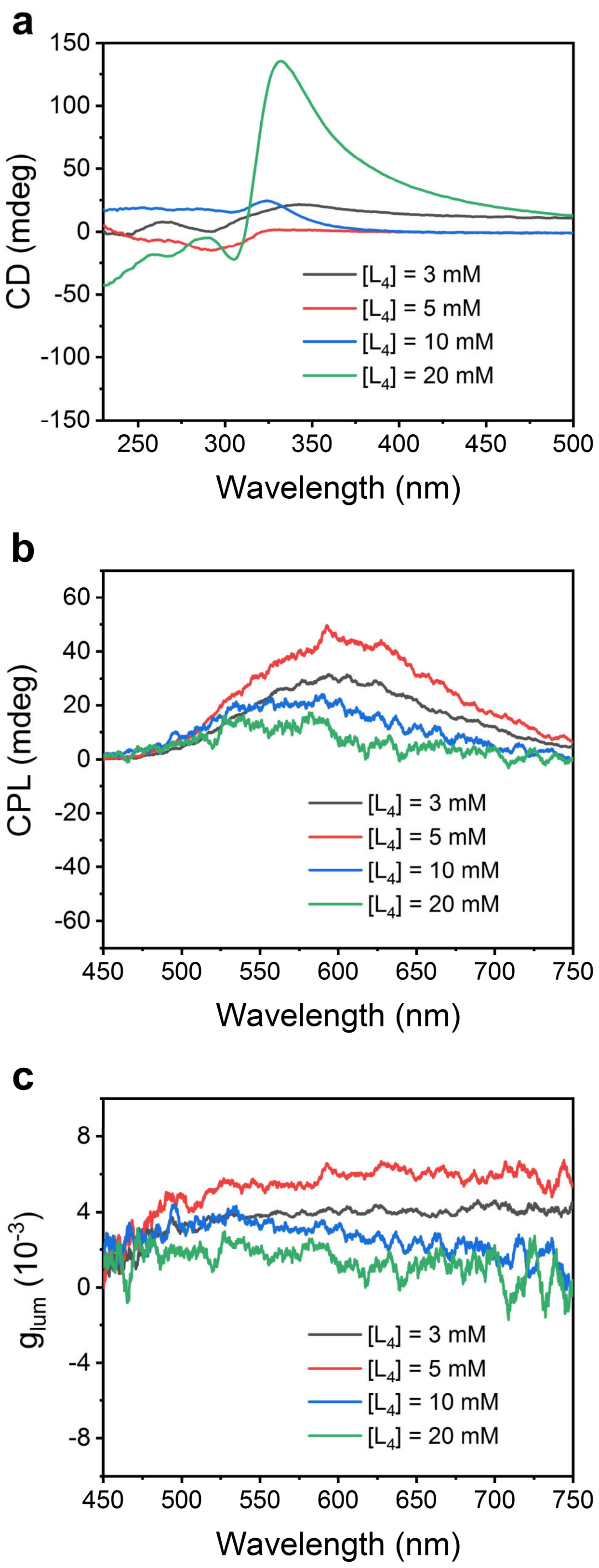

Supplementary Fig. 31. Chiroptical properties of $Cu_5I_7(L_4)_2$ hierarchically chiral polycrystals with different concentrations of L_4. a CD spectra. **b** CPL spectra. **c** g_{lum} spectra. Source data are provided as a Source Data file.

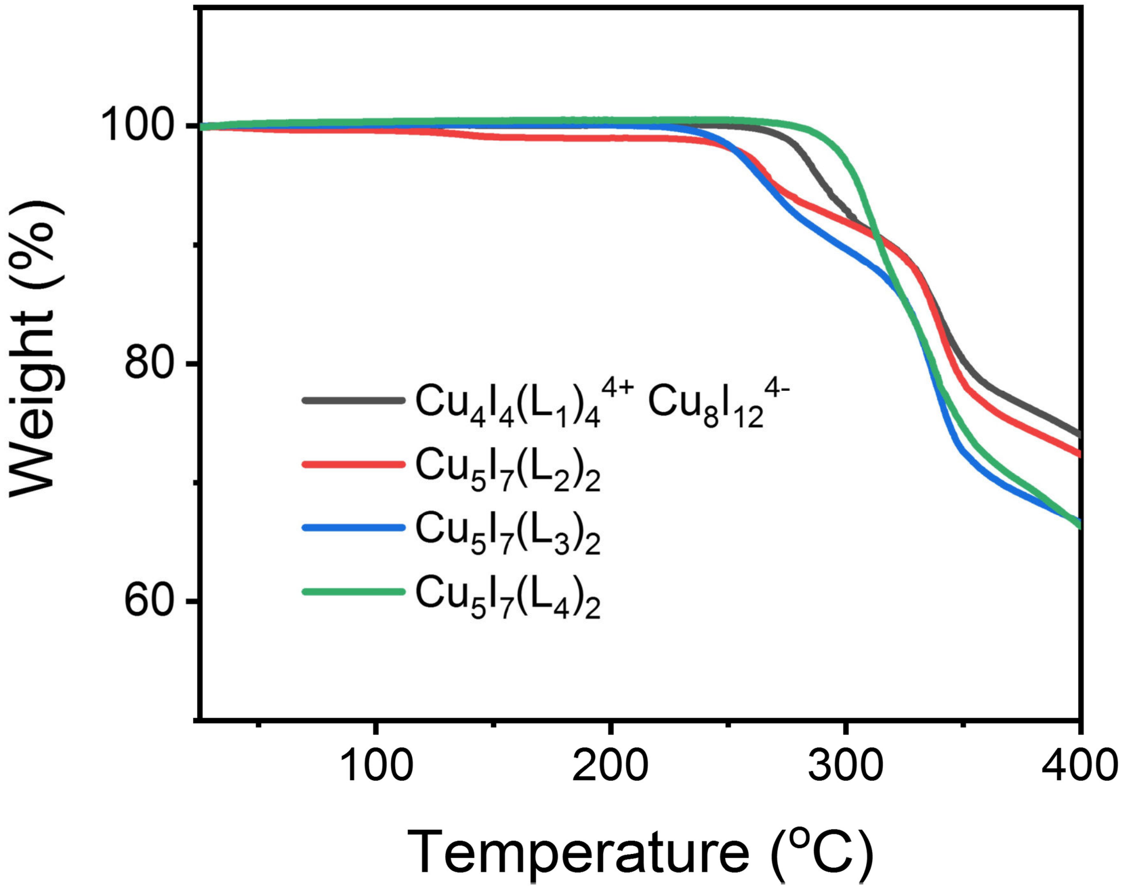

Supplementary Fig. 32. TGA curves of $Cu_4I_4(L_1)_4^{4+}$ $Cu_8I_{12}^{4-}$, $Cu_5I_7(L_2)_2$, $Cu_5I_7(L_3)_2$, and $Cu_5I_7(L_4)_2$ hierarchically structured polycrystals. Source data are provided as a Source Data file.

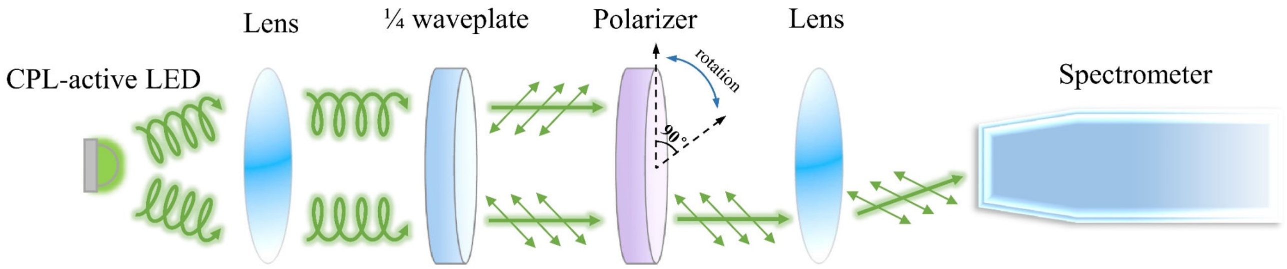

Supplementary Fig. 33. Schematic illustration of LED polarization measurement system. We measured the circularly polarized light emitted by the fabricated LED device by dividing the emitted light into left- and right-handed portions, respectively, using a quarter-wave plate and a linear polarizer to eliminate the influence of linear polarized light.

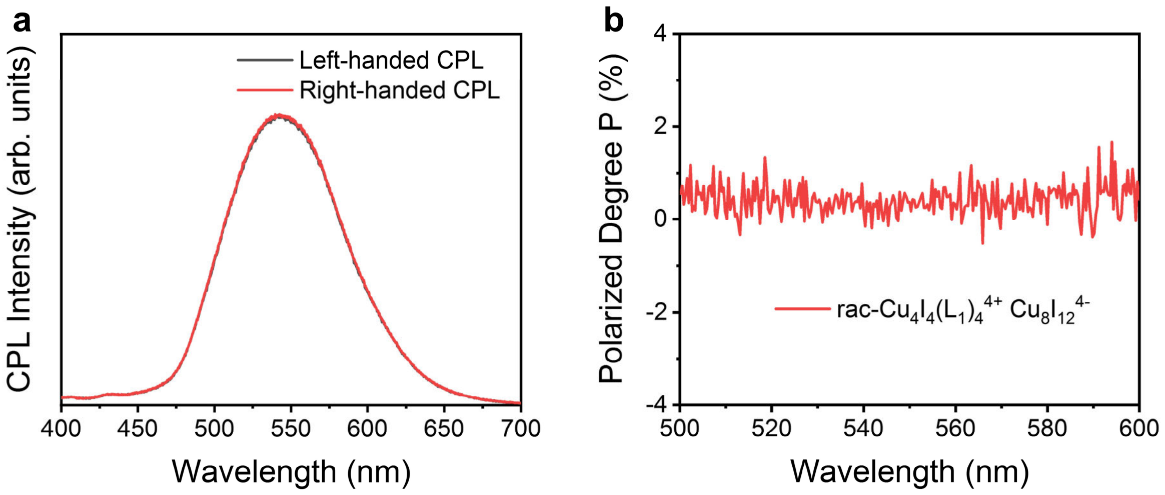

Supplementary Fig. 34. Circularly polarized characteristics of the fabricated LED device based on racemic $Cu_4I_4(L_1)_4^{4+}$ $Cu_8I_{12}^{4-}$ polycrystals powered by a 2.4 V electric source. a CPL spectra. **b** The polarized degree analysis. Source data are provided as a Source Data file.

Supplementary Note 2. The crystallization process of $Cu_5I_7(L_4)_2$ polycrystals. As shown in Supplementary Fig. 20e and f, in the beginning, $Cu_5I_7(L_4)_2$ clusters aggregated into conglobate particles, and then grew into nanosheets. PXRD patterns of nanoparticles and nanosheets show wide width of diffraction peaks, indicating low crystallinity of $Cu_5I_7(L_4)_2$ at this stage (Supplementary Fig. 18d). Additionally, a strong diffraction peak at 5° does not belong to the phase of $Cu_5I_7(L_4)_2$. According to Bragg's law, we speculate that it is owing to the two-dimensional layered structure with a large distance between two layers due to the long alkyl chain length of L_4, which gives rise to the peak at a small degree of 5°. But, as the reaction time prolonged, nanosheets were avulsed, restructured, and twisted into M-helical nanobelts (Supplementary Fig. 20g, h). And the helicity of nanobelts can also be verified by transmission electron microscopy (TEM) images (Supplementary Fig. 21). Correspondingly, the diffraction peak at 5° in the PXRD pattern is disappeared at this time, indicating the disappearance of two-dimensional structures and the transformation of nanosheets to nanobelts (Supplementary Fig. 18d).

Supplementary Table 1. Crystallography parameters of $Cu_4I_4(L_1)_4^{4+}$ $Cu_8I_{12}^{4-}$, $Cu_5I_7(L_2)_2$, $Cu_5I_7(L_3)_2$ and $Cu_5I_7(L_4)_2$ single crystals.

**Compounds**	**$Cu_4I_4(L_1)_4^{4+}$ $Cu_8I_{12}^{4-}$**	**$Cu_5I_7(L_2)_2$**	**$Cu_5I_7(L_3)_2$**	**$Cu_5I_7(L_4)_2$**
Empirical formula	$C_{22}H_{45}Cu_6I_8N_4$	$C_{24}H_{48}Cu_5I_7N_6$	$C_{22}H_{46}Cu_5I_7N_4$	$C_{26}H_{54}Cu_5I_7N_4$
Formula weight	1762.06	1626.68	1572.63	1628.73
Temperature/K	100.15	100.00(10)	100.00(10)	100.00(10)
Crystal system	tetragonal	triclinic	trigonal	triclinic
Space group	$P4_2$	$P1$	$P3_221$	$P1$
a/Å	22.0722(2)	8.69065(18)	12.1489(5)	11.5081(2)
b/Å	22.0722(2)	13.3957(3)	12.1489(5)	11.6450(3)
c/Å	8.55280(10)	18.0042(5)	22.9488(9)	16.9677(4)
α/°	90	89.407(2)	90	109.679(2)
β/°	90	89.889(2)	90	95.9038(16)
γ/°	90	79.2342(19)	120	98.0117(19)
Volume/Å^3	4166.77(9)	2059.01(9)	2933.4(3)	2092.61(9)
Z	4	2	3	2
ρ_{calc}g/cm^3	2.809	2.624	2.671	2.585
μ/mm^{-1}	50.026	7.813	8.220	7.686
F(000)	3212.0	1500.0	2166.0	1508.0
Final R indexes [I>=2σ (I)]	R_1 = 0.0615<br>wR_2 = 0.1539	R_1 = 0.0355<br>wR_2 = 0.0806	R_1 = 0.0346<br>wR_2 = 0.0637	R_1 = 0.0307<br>wR_2 = 0.0719
Final R indexes [all data]	R_1 = 0.0679<br>wR_2 = 0.1607	R_1 = 0.0392<br>wR_2 = 0.0834	R_1 = 0.0362<br>wR_2 = 0.0647	R_1 = 0.0315<br>wR_2 = 0.0724
Flack parameter	0.05(2)	0.00(3)	0.04(3)	0.056(12)
Melting point/°C	212-215	189-191	201-203	189-191

Supplementary Table 2. Summary of the photoluminescence quantum yield (PLQY) and luminescence dissymmetry factor (g_{lum}) of previously reported CPL materials.

Material types	CPL Material	Peak Wavelength (nm)	PLQY (%)	$\|g_{lum}\|$	Ref.
Clusters	$(R\text{-MBA})_4Cu_4I_4$	630	52.8	1.0×10^{-2}	1
	$(S\text{-MBA})_4Cu_4I_4$	630	59.7	6×10^{-3}	
	$[Cu_3(R\text{-BINAP})_3CO_3](^tBuSO_3)$	605	1.2	2×10^{-2}	2
	$[Cu_3(S\text{-BINAP})_3CO_3](^tBuSO_3)$	605	1.2	2×10^{-2}	
	Ag_6PD_6	575	95	4.42×10^{-3}	3
	Ag_6PL_6	575	95	4.42×10^{-3}	
	$[D\text{-valinol(18-crown-6)}]^+[Cu_5(S^tBu)6]^-$	660	47.4	9.77×10^{-3}	4
	$[L\text{-valinol(18-crown-6)}]^+[Cu_5(S^tBu)6]^-$	660	46.5	9.77×10^{-3}	
Coordination compounds	L-Cysteine capped $CaAl_2O_4$:Eu,Nd	440	-	2.1×10^{-2}	5
	D-Cysteine capped $CaAl_2O_4$:Eu,Nd	440	-	1.9×10^{-2}	
	$[Au(NHC)_2][Au(CN)_2]$ (RC-A)	500	65	1.6×10^{-3}	6
	$[Au(NHC)_2][Au(CN)_2]$ (SC-A)	500	65	1.45×10^{-3}	
	$CsEu(hfbc)_4$	595	3	1.38	7
Organic molecules	P-2-aza[4]helicene	402	-	8.7×10^{-3}	8
	M-2-aza[4]helicene	402	-	9.5×10^{-3}	
	Helical carbazole-based BODIPY analogues	411~532	20.0~36.4	7×10^{-4}~1.9×10^{-3}	9
	g-BNMe$_2$-Cp	525	53	4.24×10^{-3}	10
	m-BNMe$_2$-Cp	487	33	-	
Nanocrystals	R-α-octylamine modified $CsPbBr_3$ NCs	520	60-70	7×10^{-3}	11
	S-α-octylamine modified $CsPbBr_3$ NCs	520	60-70	6.5×10^{-3}	
	LGAM capped $CsPbBr_3$ NCs	511	47.4	7.3×10^{-3}	12
	DGAM capped $CsPbBr_3$ NCs	511	47.4	7.3×10^{-3}	
	D-cysteine-CdSe/CdS NCs	614	20	5.17×10^{-4}	13
Assemblies	LPF/DPF-2An co-gel	500	18.5	4.2×10^{-3}	14
	R-Py/TCNB	508	5.2	1.7×10^{-2}	15
	L/D-NDI-GE π-nanotube	546	-	7×10^{-2}	16
	Au-L-Cys twisted ribbons	620	-	2×10^{-3}	17
	Au-D-Cys twisted ribbons	620	-	2×10^{-3}	
	$NDI^{\bullet-}$ radical anion gels	560-700	-	0.26-0.20	18
**This work**	**$Cu_4I_4(L_1)_4^{4+}$ $Cu_8I_{12}^{4-}$ polycrystals**	**544**	**32**	**1.5×10^{-2}**	
	**$Cu_5I_7(L_2)_2$ polycrystals**	**580**	**50**	**4.0×10^{-3}**	
	**$Cu_5I_7(L_3)_2$ polycrystals**	**590**	**7**	**3.7×10^{-3}**	
	**$Cu_5I_7(L_4)_2$ polycrystals**	**620**	**6**	**6.0×10^{-3}**	

References.

1. Yao, L. *et al.* Circularly polarized luminescence from chiral tetranuclear copper(I) iodide clusters. *J. Phys. Chem. Lett.* **11**, 1255-1260 (2020).
2. Kong, Y.-J. *et al.* Photoresponsive Propeller-like Chiral AIE Copper(I) Clusters. *Angew. Chem. Int. Ed.* **59**, 5336-5340 (2020).
3. Han, Z. *et al.* Ultrastable atomically precise chiral silver clusters with more than 95% quantum efficiency. *Sci. Adv.* **6**, eaay0107 (2020).
4. Jin, Y. *et al.* Cations controlling the chiral assembly of luminescent atomically precise copper (I) clusters. *Angew. Chem. Int. Ed.* **131**, 12271-12276 (2019).
5. Hao, W., Li, Y. & Liu, M. Endowing phosphor materials with long-afterglow circularly polarized phosphorescence via ball milling. *Adv. Opt. Mater.*, 2100452 (2021).
6. Yang, J. G. *et al.* Controlling metallophilic interactions in chiral gold (I) double salts towards excitation wavelength-tunable circularly polarized luminescence. *Angew. Chem. Int. Ed.* **59**, 6915-6922 (2020).
7. Zinna, F., Giovanella, U. & Bari, L. D. Highly circularly polarized electroluminescence from a chiral europium complex. *Adv. Mater.* **27**, 1791-1795 (2015).
8. Wang, F., Gan, F., Shen, C. & Qiu, H. Amplifiable symmetry breaking in aggregates of vibrating helical molecules. *J. Am. Chem. Soc.* **142**, 16167-16172 (2020).
9. Wade, J. *et al.* 500-Fold amplification of small molecule circularly polarised luminescence through circularly polarised FRET. *Angew. Chem. Int. Ed.* **60**, 222-227 (2021).
10. Zhang, M.-Y. *et al.* Solid-state emissive triarylborane-based [2.2] paracyclophanes displaying circularly polarized luminescence and thermally activated delayed fluorescence. *Org. Lett.* **20**, 6868-6871 (2018).
11. Chen, W. *et al.* Two-photon absorption-based upconverted circularly polarized luminescence generated in chiral perovskite nanocrystals. *J. Phys. Chem. Lett.* **10**, 3290-3295 (2019).
12. Shi, Y., Duan, P., Huo, S., Li, Y. & Liu, M. Endowing perovskite nanocrystals with circularly polarized luminescence. *Adv. Mater.* **30**, 1705011 (2018).
13. Hao, J. *et al.* Ligand-induced chirality in asymmetric CdSe/CdS nanostructures: a close look at chiral tadpoles. *ACS Nano* **14**, 10346-10358 (2020).
14. Yang, L. *et al.* Highly efficient full-color and white circularly polarized luminescent nanoassemblies and their performance in light emitting devices. *Nanoscale* **12**, 6233-6238 (2020).
15. Han, J. *et al.* Enhanced circularly polarized luminescence in emissive charge-transfer complexes. *Angew. Chem. Int. Ed.* **131**, 7087-7093 (2019).
16. Wang, Y. *et al.* Bamboo-like π-nanotube with tunable helicity and circularly polarized luminescence. *Angew. Chem. Int. Ed.* **133**, 16751-16757 (2021).
17. Jiang, W. *et al.* Emergence of complexity inhierarchically organized chiral particles. *Science* **368**, 642-648 (2020).
18. Wang, Y., Jiang, Y. Q., Zhu, X. F. & Liu, M. H. Significantly boosted and inversed circularly polarized luminescence from photogenerated radical anions in dipeptide naphthalenediimide assemblies. *J. Phys. Chem. Lett.* **10**, 5861-5867 (2019).
